# Cocreation and codesign in maternal health: processes, outcomes and implementation challenges

**DOI:** 10.1136/bmjqs-2025-019030

**Published:** 2026-02-20

**Authors:** Mohamed Rishard, Kavinda Rajaratne, Kavinda Jayasingha, Hemantha Senanayake, Millawage Supun Dilara Wijesinghe

**Affiliations:** 1 Department of Obstetrics and Gynaecology, Faculty of Medicine, University of Colombo, Colombo, Sri Lanka; 2 University Teaching Unit, De Soysa Hospital For Women, Colombo, Western, Sri Lanka; 3 Health Promotion Bureau, Colombo, Western, Sri Lanka

**Keywords:** Healthcare quality improvement, Health policy, Implementation science

## Abstract

**Objective:**

To describe the application of cocreation/codesign in maternal health, characterise reported processes (including barriers/facilitators) and summarise clinical and experiential outcomes without inferring causal effects.

**Methods:**

Following the Preferred Reporting Items for Systematic Reviews and Meta-Analyses guidelines, a comprehensive search of PubMed, MEDLINE, Embase, Cochrane Library and ScienceDirect (2014–2025) identified 1487 records. After deduplication and screening, 60 studies were included in this review. The eligibility criteria encompassed qualitative and quantitative studies employing cocreation or codesign in maternal or obstetric care, with open-access English full texts. Two blinded reviewers screened the studies using Rayyan and resolved conflicts by consensus agreement. Data extraction followed the Cochrane guidelines, and a narrative synthesis was performed because of study heterogeneity. The risk of bias was assessed using the Newcastle-Ottawa Scale, risk-of-bias 2 and the Risk Of Bias In Non-Randomised Studies - of Exposure (ROBINS-E) tools.

**Results:**

Across 60 heterogeneous studies, cocreation and codesign were used to develop and adapt psychosocial support, communication and feedback interventions and digital tools such as decision-support and telemonitoring systems. Studies most often reported process and experiential outcomes, including acceptability, satisfaction, engagement and perceived relevance, to users and communities. The designs, contexts and outcome measures varied, and comparative evidence was limited. Common challenges included power imbalances, time and resource constraints, and difficulties in spreading and sustaining interventions. The reported enablers included early and ongoing stakeholder involvement, iterative cycles and attention to access barriers, including digital inclusion.

**Conclusion:**

When cocreation and codesign are clearly described and suited to the context, they can support culturally relevant and equitable maternal care by incorporating stakeholder voices into service and intervention development. The current evidence does not support firm conclusions regarding effectiveness. Future work should transparently report codesign methods, specify the intended mechanism and context, and evaluate codesigned interventions with stronger comparative designs and longer follow-ups.

**PROSPERO registration number:**

CRD42024622020.

**WHAT IS ALREADY KNOWN ON THIS TOPIC**

- Cocreation and codesign methodologies have been used in various healthcare fields to improve patient engagement, intervention relevance and care quality.
- While these approaches have shown promise in enhancing health outcomes, their application in maternal healthcare remains understudied.
- Existing reviews have focused on specific geographical contexts or broader public health domains rather than systematically evaluating their impact on maternal health.

**WHAT THIS STUDY ADDS**

- This systematic review synthesises evidence from 60 studies, demonstrating that cocreation and codesign methodologies are associated with improved maternal mental health, strengthened provider–client relationships and enhanced digital interventions.
- These approaches contribute to culturally relevant patient-centred maternity care by fostering stakeholder engagement and addressing systemic barriers to care.
- However, challenges such as power imbalances, resource constraints and scalability limitations remain unresolved.

**HOW THIS STUDY MIGHT AFFECT RESEARCH, PRACTICE OR POLICY**

- This study highlights the need for a standardised framework to evaluate and implement cocreation in the field of maternal health.
- Policymakers should support scalable, community-driven models, invest in digital equity and integrate stakeholder-driven training programmes to improve the quality of maternity care.
- Future research should focus on longitudinal assessments, intersectional approaches and digital innovations to enhance the global maternal health outcomes.

## Introduction

### Background

 The field of maternal health has undergone significant advancements in recent decades; however, challenges related to patient safety, care quality and maternal outcomes persist.^1^ In response, healthcare systems worldwide are increasingly adopting quality improvement (QI) strategies aimed at enhancing clinical practices, reducing maternal morbidity and mortality and ensuring safe and effective care for pregnant women. One innovative approach to improving healthcare quality is through codesign and cocreation.^2 3^

Cocreation, codesign and coproduction are often used interchangeably to describe collaborative processes involving multiple stakeholders in the development of initiatives.^4 5^ Cocreation refers to a holistic, collaborative approach in which diverse stakeholders engage in creative problem-solving across all stages of an initiative, from identifying the problem to generating solutions for its implementation and evaluation. By contrast, codesign focuses more specifically on the active collaboration of stakeholders in designing solutions to a problem that has already been identified. Both approaches emphasise inclusivity and shared ownership, ensuring that the resulting initiatives are tailored to the needs and perspectives of all participants.^4^

Cocreation fosters the participation of a wide range of stakeholders, including community members, health workers and policymakers, in public health interventions. Involving these diverse groups in planning and development can facilitate not only implementation but also sustainability and scalability of interventions.^6^ Furthermore, engaging stakeholders in the design and execution of interventions is believed to result in more contextually relevant solutions, greater satisfaction and improved adherence to recommended practices. The first examples of experience-based codesign (EBCD) in healthcare emerged from head-and-neck cancer clinics^7 8^ and were subsequently applied in various settings, such as emergency health services,^9^ cancer care,^10^ neonatal care,^11^ outpatient services for the elderly^12^ and mental health.^13 14^ In health services research, codesign activities are frequently described using ‘user-centred’ or ‘human-centred’ design (UCD/HCD) terminology. UCD is defined by ISO 9241–210 as an approach that makes systems ‘usable and useful’ by focusing on users, their needs and context through iterative cycles across the design lifecycle; as such, it provides the methodological and theoretical backbone for many codesign projects in healthcare.^5 7 15^

QI projects in obstetrics, particularly those focused on maternal health, can significantly benefit from codesigned methodologies.^6 16^ Recent studies have demonstrated the effectiveness of codesign in improving information delivery practices during pregnancy and childbirth, enhancing staff-patient communication and streamlining critical care pathways.^17^ Despite these advancements, a significant research gap remains in understanding the broader applicability, effectiveness and challenges of codesign and cocreation methodologies, specifically in obstetric and maternal care settings. Given the growing number of emerging projects in obstetrics and maternity care, a comprehensive synthesis of the international evidence base is timely and necessary. Existing systematic reviews have often been limited in scope, focusing on a specific geographical context,^18^ examining barriers and facilitators of research participation rather than co-creation per se,^19^ or addressing the effectiveness of co-creation in research across international health literature without a specific focus on maternal or obstetric care.^3^ These limitations have created a critical gap in the evidence base, particularly in understanding how cocreation and codesign can be systematically applied to improve maternal health outcomes in diverse settings.

Therefore, we set out four objectives to guide the review: first, to describe how cocreation and codesign methodologies have been applied in maternal health across settings, populations and intervention types; second, to characterise the codesign processes reported and synthesise the barriers and facilitators to their implementation; third, to summarise the reported outcomes associated with these approaches, including process, clinical and experiential outcomes (without inferring causal effects); and fourth, to identify key gaps to inform future research priorities and policy development.

Our purpose is descriptive and integrative rather than causal; we do not evaluate the effectiveness of codesign. By mapping the principles and practices of cocreation/codesign to the contexts in which they were applied, we identified plausible mechanisms through which these approaches may influence care delivery and patient experience and highlighted recurrent implementation barriers and facilitators. Taken together, this review provides a consolidated evidence base to inform the design, implementation and evaluation of future quality-improvement initiatives in obstetrics, supporting context-appropriate, user-responsive strategies and generating hypotheses for studies that can rigorously test impact and equity effects.

## Methods

This systematic review was conducted in accordance with the Preferred Reporting Items for Systematic Reviews and Meta-Analyses (PRISMA) guidelines. The protocol for this review was registered with PROSPERO (CRD42024622020). No amendments were made.

### Search strategy

A comprehensive search strategy was employed across four electronic databases: PubMed, MEDLINE, Embase and the Cochrane Library, using the following combination of keywords and Boolean operators: (‘codesign’ OR ‘co-design’ OR ‘cocreation’ OR ‘co-creation’ OR ‘participatory design’ OR ‘user-centered design’ OR ‘co-production’ OR ‘stakeholder engagement’) AND (‘obstetric care’ OR ‘maternal health’ OR ‘childbirth’ OR ‘intrapartum care’ OR ‘pregnancy’ OR ‘maternity services’ OR ‘perinatal care’). Owing to the limitations of using Boolean operators in ScienceDirect, a simplified search strategy was applied using (codesign OR cocreation OR stakeholder engagement) AND (pregnancy OR childbirth OR maternal health OR obstetric care OR intrapartum care OR maternity services) (online supplemental file 1). The reference lists of the included studies were manually reviewed to identify further relevant articles. Because our review examined how collaborative design is used to shape maternal health services/interventions, we prioritised cocreation/codesign in the analysis while still searching for coproduction to avoid missing eligible studies that used that label.

### Eligibility criteria

#### Inclusion criteria

Both qualitative and quantitative studies that used cocreation or codesign methodologies in obstetric or maternal care settings were eligible for inclusion. Only open-access full-text articles in English were considered, with studies published from 2014 until 1 January 2025.

The exclusion criteria were as follows: studies that did not involve stakeholder participation in the design process or were not specific to obstetric or maternal care. Review articles, editorials, conference abstracts, commentaries, book chapters and protocols were excluded.

### Study selection

The study selection process was conducted using a blinded approach in two steps via the semiautomated tool Rayyan, with one author (KR) as the reviewer and the other (KJ) as the collaborator. In the first round, title abstract screening and removal of duplicates were performed, with conflicts resolved by a third author (MR). Moreover, the second round also adopted a similar blinded approach for full-text screening, with conflicts resolved by the author mentioned earlier. All titles, abstracts and full texts were independently screened by two authors (KR, KJ). The study selection process followed the PRISMA 2020 flow diagram (figure 1) for updated systematic review.^20^

**Figure 1:**
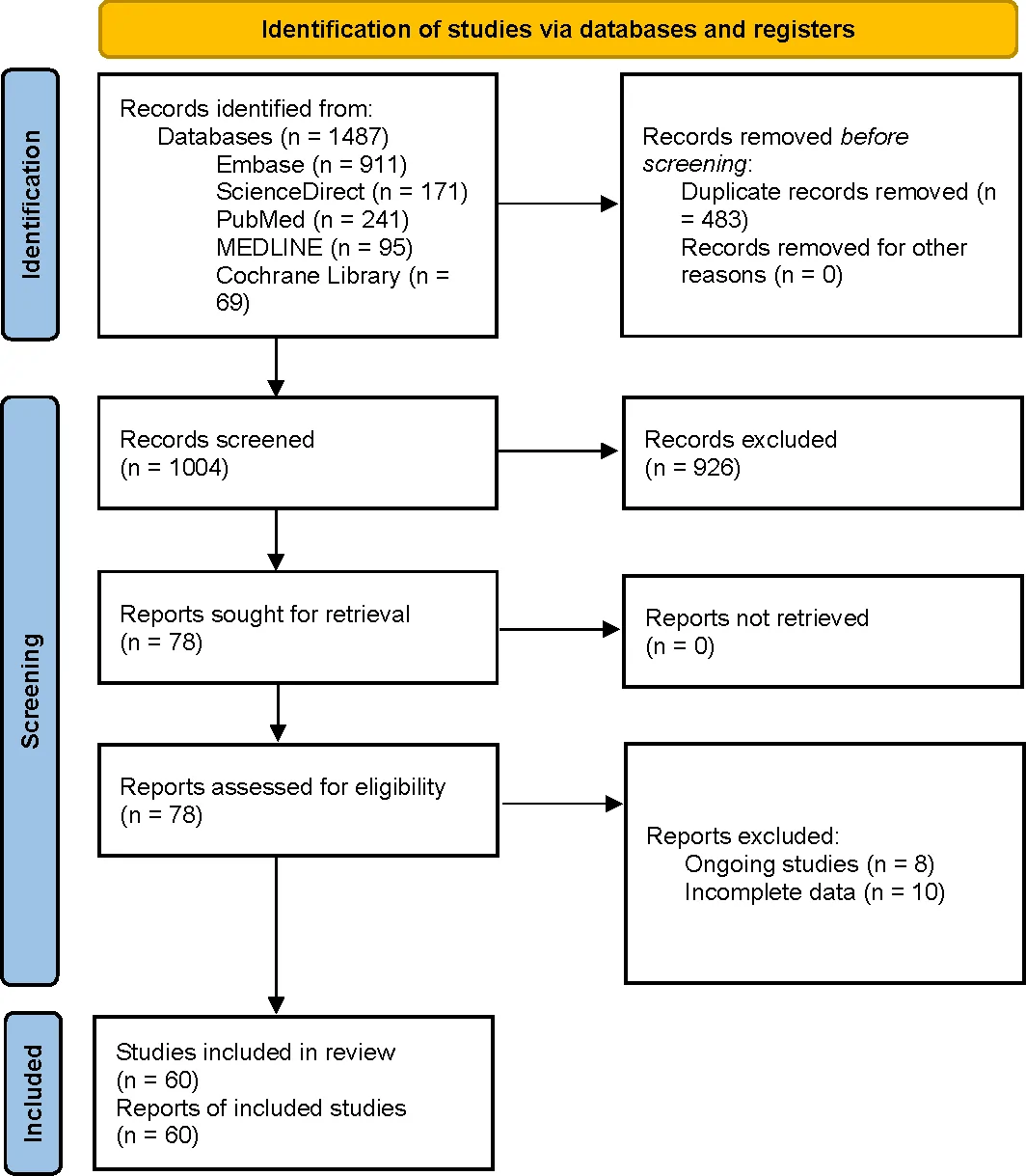
PRISMA 2020 flow diagram for study selection after initial filtering.^20^ PRISMA, Preferred Reporting Items for Systematic Reviews and Meta-Analyses.

### Data extraction

Data extraction was performed according to the Cochrane Handbook for Systematic Reviews of Interventions.^21^ The initial data extraction was conducted by one author and cross-checked by the second and third authors. Finally, the fourth author … verified data completeness. Conflicts among the authors were discussed, and discrepancies were settled (through consensus). Data extraction was systematically performed for each included study, focusing on predefined key study characteristics, including author(s), year of publication, study location, study objectives, study design, stakeholder involvement, interventions developed, challenges encountered and the overall impact of the interventions.

### Data synthesis

Given the expected heterogeneity in study designs, populations and outcomes, a narrative synthesis was performed to summarise the findings. The synthesis emphasised the reported outcomes associated with cocreation or codesign approaches in maternal health, identifying key trends, patterns and lessons learnt across the included studies. However, a meta-analysis was not conducted because of the mixed nature of the included evidence.

### Risk of bias assessment

Quality assessment of cohort and case–control studies was performed using the Newcastle-Ottawa Scale (NOS).^22^ The randomised controlled trial (RCT) quality was assessed using the Cochrane risk-of-bias (RoB2) tool.^23^ For other non-randomised studies, we used the ROBINS-E tool to examine the potential biases.^24^

## Results

The PRISMA flowchart illustrates the process of identifying and selecting studies for this systematic review (figure 1). Initially, 1487 records were identified from multiple databases, including PubMed (241), MEDLINE (95), Embase (911), ScienceDirect (171) and Cochrane Library (69). Before screening, 483 duplicate records were removed, leaving 1004 for screening. After the initial screening, 926 records were excluded from this study. These 78 reports were assessed for eligibility, resulting in the exclusion of 18 reports, 8 due to ongoing studies and 10 reporting incomplete data. Finally, 60 studies were included in this review (table 1, online supplemental file 2).

**Table 1:** Summary of included studies (n=60)

Author/country	Coapproach/framework	Participants/stakeholders	Intervention	Findings/conclusion
Isangula *et al* (Tanzania)^25^	Cocreation/codesign	MCH nurses, clients, administrators (30)	Patient-centred care promotion, awards, strengthen complaints mechanism, disciplinary measures	HCD enabled joint problem-solving and therapeutic relationship strengthening
Isangula *et al* (Tanzania)^26^	Co-design	Nurses, clients, administrators and local NGOs	Workshops developing behaviour change solutions	Co-design builds commitment and improves provider-client relations
Kenyon *et al* (UK)^27^	Co-design (EBCD)	Clinicians and service users	Revised referral and decision process for CS requests	New pathway acceptable to both staff and women
Mamani-Mategula *et al* (Malawi)^28^	Co-design (EBCD)	End-users and healthcare providers (n=40)	Mitigation strategies for REVAMP-TT trial	Co-design revealed barriers and facilitators for implementation
Jith *et al* (India)^29^	Cocreation	Local governments (PRIs), CBOs, CSOs	Community collaboration platforms	PRIs enable participation and implementation of nutrition programmes
Chomat *et al* (Guatemala)^30^	Codesign	Circle leaders (16)	Group CBT, IPT, mindfulness activities	Feasible and acceptable psychosocial support for indigenous women
Nagraj *et al* (India)^31^	Codesign (HCD)	ASHAs and primary care doctors	Mobile decision-support+training	Co-design enhanced acceptability and implementation
Yang *et al* (China)^32^	Codesign	Pregnant women & midwife	Group sessions+WeChat reminders	High acceptability and feasibility
Bogale *et al* (Palestine)^33^	Codesign	Women and providers (26 total)	Text-message ANC reminders	Co-designed messages bridged ANC gaps
Whybrow *et al* (UK)^34^	Codesign	Women with chronic hypertension and clinicians	Shared-decision tool for chronic hypertension patients	Reduced decisional conflict, improved intentions
McKellar *et al* (Australia)^35^	Codesign	Mothers, midwives, technology designers	Digital mental-health tracking tool	Useful for self-monitoring and help-seeking
Wright *et al* (NZ)^36^	Codesign	Midwives, physicians, administrators	MatCHAT decision-support system	Feasible but contextual barriers exist
Fair *et al* (UK)^37^	Cocreation/codesign	Healthcare providers and service users	Survey+stakeholder feedback	Need for more services and clear communication
Ellis *et al* (Australia)^38^	Codesign	Peer workers and health staff	Online module for perinatal ED	Improved knowledge and confidence
Carlin *et al* (Australia)^39^	Codesign	Aboriginal women and healthcare providers	KMMS screening tool	Culturally appropriate screening possible
Osaki *et al* (Tanzania)^40^	Cocreation	Frontline providers and experts	Locally adapted PartoMa clinical guidelines	Engagement and reflexivity improved uptake
Stewart *et al* (Gambia)^41^	Cocreation/codesign	Women, midwives, CBCs, Kanyelengs and griots	Music-based mental health intervention	Cross-disciplinary partnerships enhance cultural fit
AlJaberi (USA)^42^	Codesign	Low-income transmigrant women+healthcare experts	mHealth pregnancy platform	Highlighted social sharing needs and gaps
Ba *et al* (Mali)^43^	Codesign	Midwives, mothers, designers, MOH representatives	MMS branding and counselling package	High acceptability; guides local adaptation
Feroz *et al* (Pakistan)^44^	Codesign	Pregnant women, clinicians, healthcare providers, digital health experts	Mobile telemonitoring programme	Feasible solution for high-risk pregnancies
Jenkinson *et al* (Australia)^45^	Codesign (PDSA)	Consumers, midwives, healthcare experts, legal experts	Guideline and support resources	First formal framework for choice-enabling care
Molina *et al* (USA)^46^	Codesign	Clinicians and Spanish-speaking patients	Communication tool	Enhanced trust and cross-language engagement
Sharma *et al* (UK)^47^	Coproduction	Local service users and healthcare providers	Community education intervention	Feasible and acceptable approach to ANC timing
Hall *et al* (UK)^48^	Codesign (person-based)	Mental health experts and mothers	Mindfulness and nature activities	Tailored programme improved mood and well-being
Mancinelli *et al* (Italy)^49^	Codesign	Pregnant women and psychologists	e-Learning modules (guided vs unguided)	Interest high but unguided model unfeasible
Venkateswaran *et al* (Bangladesh)^50^	Codesign	End users (2016–2018)	Digital registry with decision support	Reduced perinatal mortality but implementation issues
Auxier *et al* (Finland)^51^	Cocreation	Pregnant women, nurses, technology engineers	ŌURA smart ring use	Co-creation aided personalised eHealth
Sequeira D’mello *et al* (Tanzania)^52^	Cocreation (AHEAD model)	Postnatal women and healthcare staff, public health experts	Respectful Maternity Care tool	Contextualised and validated for routine use
Murugesu *et al* (Netherlands)^53^	Cocreation (IM+UCD)	Healthcare providers and clients	Conversation aid+training	Supports HL-sensitive SDM but time-intensive
Blomgren *et al* (Uganda)^54^	Cocreation (CFIR)	Midwives, policy makers, doctors, recent mothers	Peer-learning and education materials	Strengthened midwifery practice and collaboration
Metta *et al* (Tanzania)^55^	Codesign	Postpartum women, healthcare staff	Input for QI intervention	Empathic care low; mistreatment normalised
Kildea *et al* (Australia)^56^	Codesign	First Nations community and services	Birthing on country model	Improved maternal and infant outcomes
Silver *et al* (Canada)^57^	Codesign (Joyce’s principle)	Inuit & Cree participants+perinatal providers	Cultural safety training and supports	Enhanced cultural safety and family presence
Fietta *et al* (Italy)^58^	Codesign	Pregnant women, women with breast cancer, psychologists, clinicians	Mobile stress-management app	Usable and supportive digital tool
Meloncelli *et al* (Australia)^59^	Codesign (COM-B/BCW)	Researchers+women with GDM	Dietary education and enablement intervention	Clear and replicable complex intervention
Mndala *et al* (Malawi)^60^	Cocreation	Healthcare staff, district officers, patients	Updated health passport book	Improved documentation and care quality
Hunter *et al* (Africa, SE Asia, EMRO regions)^61^	Codesign	Midwifery experts (Europe)	Education assessment tool (MATE)	Culturally relevant tool for midwifery education
Harris *et al* (UK)^62^	Codesign	Midwives and midwifery educators	Educational training package	Helpful but needs further adaptation
Mancinelli *et al* (Italy)^63^	Codesign	Pregnant women, psychologists, digital health developers	Behavioural activation chatbot	Need balance between tech and human support
Musie *et al* (S. Africa)^64^	Cocreation (SPO model)	Maternity care experts and TBAs	Collaboration framework	Framework guides policy and practice improvement
Bitar *et al* (Sweden)^65^	Codesign (five phases)	Multidisciplinary team and users	Multimodal communication app	Need for cultural sensitivity and usability
Amoakoh *et al* (Ghana)^66^	Cocreation (EBCD/PRODUCES)	120 stakeholders (policy, providers, patients, survivors of HDP)	Risk communication and SDM toolkit	Supports clinical and experiential quality of care
Álvarez-Pérez *et al* (Belgium, Denmark, Italy, Spain, Sweden)^67^	Codesign (CoPs)	Pregnant and lactating women, midwives, lactation experts	Online MOOCs	Improved self-perceived digital literacy
Merriel *et al* (UK)^68^	Codesign	Postnatal and antenatal women, midwives, clinicians, psychologists	Structured ANE session	Deliverable and positively received
Waddell *et al* (Australia)^69^	Codesign	Clinicians, health service leaders, patients, policy makers	SDM training and patient portal	Guides policy-to-practice transition
Hanrahan *et al* (UK, Ireland)^70^	Codesign (mixed methods)	Healthcare professionals and service users	ENCOUNTER app	Acceptable behaviour-change tool
Melwania *et al* (Tasmania)^71^	Codesign (workshops)	Public health and maternal health professionals	System and policy recommendations	Empowers women and informs policy
Keedle *et al* (Australia)^72^	Codesign	Service users and maternity organisations	Cross-sectional online survey	Data to inform service design
Salmon *et al* (UK)^73^	Codesign	Pregnant women, recent mothers, public health providers, healthcare professionals, policy actors	Physical activity resources	Collaborative methods improve relevance
Borbolla Foster *et al* (Australia)^74^	Codesign (action process)	Consumers and multidisciplinary team	Population-based first-trimester pathway	Aligned care model reduced risks
Wiseman *et al* (UK)^75^	Codesign	Midwives, pregnant and postnatal women	Group ANC model (pregnancy circles)	Valued relationships; implementation challenges
Haith-Cooper *et al* (UK)^76^	Codesign	Midwives and postnatal women	3 min animated video	Feasible educational tool
Parsons *et al* (UK)^77^	Codesign (IM)	Pregnant women, healthcare providers, public health professionals	Animated risk communication tool	Implemented in UK campaigns
Christiansen *et al* (Denmark)^78^	Cocreation+BCW	Postpartum parents, healthcare professionals, IT experts	App with videos and weight tracking with notifications	Met user needs and supported behaviour change
Blewitt *et al* (Australia)^79^	Codesign (IM protocol)	Community-service employees (women 18–45 years) and project staff	HiPPP Portal—digital workplace programme for healthy lifestyle	Feasible and beneficial; IM offers a structured roadmap for collaborative design
Guyatt *et al* (Australia)^80^	Codesign (CFIR framework)	Expectant and new parents, healthcare providers	Comprehensive perinatal education programme	Framework provides guidance for theory-based implementation in maternity care
Klingberg-Allvin *et al* (East & Southern Africa)^81^	Cocreation (workshops)	Researchers, health ministry representatives, FIGO experts, NGOs, media partners	Regional workshops and training curricula	Built capacity and networks; co-creation vital for sustainable CAC delivery
Bosak *et al* (USA)^82^	Codesign (adapted EBCD)	Women with lived experience, SUD treatment providers	Input for residential treatment programme design	Highlighted structural and relational barriers; emphasised trauma-informed care
Harrison *et al* (Australia)^83^	Cocreation (RCT design)	13 pregnant women with GDM	Cocreated infographic+standard education	Improved short-term knowledge and self-efficacy for exercise
Meloncelli (Australia)^84^	Codesign (PAR+MRC guidelines)	Women with GDM experience, researchers	Dietary intervention—healthy gut diet	Reproducible and adaptable intervention per TIDieR guidelines

ANC, antenatal care; ANE, antenatal education; ASHA, Accredited Social Health Activists; BA, behavioural activation; BCW, behaviour change wheel; CAC, comprehensive abortion care; CBC, community birth companions; CBO, community-based organisations; CBT, cognitive–behavioural therapy; CFIR, Consolidated Framework for Implementation Research; CHW, community health workers; COM-B, capability, opportunity, motivation—behaviour; CoP, communities of practice; CS, caesarean section; CSO, Civil Society Organizations; DAISI, Digital Animation In Service Improvement; EBCD, experience-based co-design; ED, eating disorder; GDM, gestational diabetes mellitus; HCD, human-centred design; HiPPP, health in planning, pregnancy and postpartum; HL, health literacy; ICT, Information and Communication Technology; IM, intervention mapping; IMAP, initial maternity assessment and planning; IPT, interpersonal therapy; KMMS, Kimberley Mum’s Mood Scale; MATE, Midwifery Assessment Tool for Education; MCH, maternal and child health; MMS, multiple micronutrient supplementation; MOH, Ministry Of Health; MOOC, Massive Open Online Courses; MRC, Medical Research Council; NGO, non-governmental organisation; PAR, participatory action research; PDSA, Plan-Do-Study-Act; PFMT, pelvic floor muscle training; PPI, Patient and Public Involvement; PRIs, Panchayat Raj Institutions; RCT, randomised controlled trial; RMC-T, Respectful Maternity Care Tool; SDM, shared decision-making; SPO, structure-process-outcome; SPOT, severe pre-eclampsia adverse outcome triage; SUD, substance use disorder; TBA, traditional birth attendants; TCC, targeted client communication.

This systematic review examined a diverse range of cocreation and codesign interventions for maternal health, across multiple settings. The included studies employed HCD,^25 26^ EBCD^27 28^ and stakeholder-driven participatory methodologies to develop interventions aimed at enhancing maternal mental health, antenatal care (ANC), midwifery education, respectful maternity care (RMC) and digital health solutions. The authors commonly reported stakeholder engagement, service delivery changes and patient-reported benefits (table 1, online supplemental file 2).

Our review included studies spanning a broad mix of designs: RCTs (n=5), pilot/feasibility trials (n=8), mixed-methods (n=12), qualitative/ethnographic (n=15) and development/implementation studies (n=20). Geographically, evidence came from Africa (n=14), Asia (n=12), Europe (n=10), Australasia (n=8), North America (n=9) and Latin America (n=7), with particularly high representation from Tanzania, India and Australia. Aims clustered around maternal mental health (n=12), digital innovation/decision support (n=15), RMC/shared decision-making (SDM) (n=10), ANC/nutrition (n=8), midwifery education/QI (n=5) and broader maternal outcomes (n=10), illustrated by interventions such as the JuNEX app (Italy), Respectful Maternity Care Tool (RMC-T) (Tanzania) and Kimberley Mum’s Mood Scale (KMMS) (Australia). Across this corpus, authors most often reported process outcomes (eg, stakeholder engagement, acceptability and feasibility), followed by clinical/service indicators (eg, ANC attendance, hypertension screening, perinatal outcomes) and patient-reported experiences (eg, mental health, satisfaction, empowerment), including improved screening rates with KMMS and reduced decisional conflict in a UK trial.

### Applications of cocreation and codesign in maternal health

This review shows that codesign approaches in pregnancy and obstetric care have been applied in implementation and intervention studies that have used collaborative methods to improve maternal health outcomes. Many studies have refined and tested interventions that integrate UCD with evidence-based practice. For example, Jith *et al*^29^ examined evidence-based nutritional interventions for pregnant women in Kerala, emphasising the importance of embedding the local context into intervention planning to enhance the effectiveness of maternal nutrition programmes.

RCTs are frequently used to evaluate codesigned obstetric care interventions. Chomat *et al*^30^ used an RCT to test women’s circles for mental health within pregnancy care. As the field standard for estimating intervention impact, RCTs provide robust evidence of the effectiveness of cocreated solutions for improving maternal health. RCTs by Nagraj *et al* and Yang *et al* further assessed digital tools and behavioural interventions in maternal health, illustrating the versatility of codesign in contemporary healthcare settings.^31 32^

Developmental and implementation work focuses on building and testing these interventions. Bogale *et al*^33^ used a participatory approach to develop tools for electronic health registries for pregnant women in Palestine. Such studies evaluate feasibility and acceptability and consider scalability across health-system contexts. A multisite NHS implementation of a SDM intervention for women with chronic hypertension used mixed methods and the RE-AIM framework to assess reach, effectiveness and acceptability, highlighting the centrality of stakeholder engagement in complex maternity care interventions.^34^

Pilot studies also help establish the feasibility of codesigned approaches in pregnancy and obstetric care. McKellar *et al*^35^ piloted a codesigned digital mental-wellbeing tool for pregnant women to ensure alignment with user needs. Isangula *et al*^25^ reported pilot work aimed at strengthening provider–client relationships in maternal healthcare, spanning the design and testing phases before a scale-up. The MatCHAT study used a mixed-methods feasibility design to evaluate a digital screening and clinical decision-support tool for midwives, identify contextual implementation barriers and emphasise the role of digital innovation in perinatal care.^36^

Mixed-method designs are also common in this field. Fair *et al*^37^ combined qualitative and quantitative approaches to examine healthy weight services during pregnancy, providing insights into both development processes and measurable outcomes. Ellis *et al*^38^ used a pre–post design to evaluate an online education module for managing peripartum eating disorders, tracking changes in knowledge and behaviour. A convergent mixed-methods study on self-monitoring eHealth systems blended wearables with interviews to show how pregnant women adapt to digital health tools, framing access as an evolving co-creation process. KMMS implementation similarly used a comprehensive mixed-methods strategy to scale a culturally adapted perinatal screening tool.^39^

Codesign is prominent in ethnographic and qualitative work, where adaptation to culture and context is central to the process of design. Osaki *et al*^40^ applied ethnographic methods to tailor interventions to specific cultural settings, and Stewart *et al*^41^ used qualitative methods to explore the contribution of social singing to perinatal mental health in Gambia. A participatory action research study with Caribbean transmigrant women in the USA investigated mHealth technologies for pregnancy well-being, emphasising culturally relevant social and entertainment features.^42^ In Bamako, Mali, a qualitative study assessed micronutrient supplementation strategies, highlighting stakeholder-led adaptations to improve adherence.^43^ In Pakistan, an interpretive qualitative study explored telemonitoring preferences among high-risk pregnant women, reinforcing the need for a contextualised design in low-income and middle-income countries.^44^ Participatory research in Queensland, Australia, supported respectful care guidelines for women who declined the recommended maternity services.^45^

Design-based approaches, such as HCD, have been used to address language and trust barriers in pregnancy care. One study cocreated a communication tool with Spanish-speaking patients and clinicians, adapting the ‘The Five Habits’ model to maternity care.^46^ A UK community-based intervention combined coproduction with implementation science to improve timely ANC initiation among diverse low-income populations, demonstrating the importance of equity-driven codesign.^47^

### Reported outcomes associated with codesign

#### Maternal mental health

Cocreation methods have been widely used to develop culturally appropriate and context-specific mental health interventions for mothers. In Guatemala, Chomat *et al*^30^ implemented the Women’s Circles Program, a psychosocial support model involving Indigenous women, community health workers and maternal health experts to promote peer support, emotional well-being and resilience during economic hardship and social isolation. In England, Hall *et al*^48^ designed a nature-based intervention featuring guided outdoor activities, sensory meditation and mindfulness-based exercise to support postnatal mental health in mothers. Mancinelli *et al*^49^ introduced JuNEX, a chatbot-based digital behavioural-activation tool codesigned with perinatal mental health specialists and expectant mothers in Italy; reported benefits included reductions in maternal anxiety, improved emotional self-regulation and greater engagement with ANC. In New Zealand, Wright *et al*^36^ developed MatCHAT via codesign with midwives and mental health professionals to integrate e-screening and decision support for depression, anxiety, substance use and family violence screenings. Although well received, implementation has been limited by workforce and infrastructure constraints in the past. In Western Australia, Carlin *et al*^39^ implemented a KMMS codesigned by Aboriginal women and observed increased perinatal mental health screening and more comprehensive identification of concerns in remote settings.

#### Strengthening healthcare provider–client relationships

Improving provider–client relationships is another major focus. In Tanzania, Isangula *et al*^25^ used a HCD to identify communication challenges, provider attitudes and patient satisfaction issues through stakeholder workshops. Codesigned responses included recognition and incentive programmes for nurses, complaints/feedback mechanisms for patients and patient-centred care training for providers. The findings indicated improved trust, higher satisfaction and better adherence to ANC protocols, reinforcing the value of patient–provider collaboration.

#### Digital innovations and decision-support systems

Cocreated digital solutions are becoming increasingly prevalent. In Bangladesh, Venkateswaran *et al*^50^ developed an eRegistry codesigned by midwives, obstetricians and policymakers, integrating clinical decision support, targeted client communication and a facility dashboard to improve maternal health. Reported improvements included timelier ANC attendance and hypertension screening as well as technical integration and data sharing challenges. In India, Nagraj *et al*^31^. co-developed SMARThealth Pregnancy, a mobile clinical decision-support system for community health workers managing high-risk pregnancies, with reported gains in early risk detection, ANC visits and patient–provider interactions. In the USA, AlJaberi^42^ explored mHealth codesigned with low-income Caribbean transmigrant women, highlighting the need for social support and entertainment features to reduce isolation and increase engagement. Auxier *et al*^51^ studied cocreation using a perinatal eHealth self-monitoring system in Finland. Access to wearables and personalised dashboards supported adaptive self-care behaviours, with language preferences highlighting the importance of personalisation and linguistic accessibility. In Pakistan, Feroz *et al*^44^ examined user needs for a telemonitoring programme for high-risk pregnancies, with patients and providers prioritising real-time data sharing, automated guidance and language-specific interfaces; enthusiasm was tempered by digital literacy and device accuracy concerns. In New Zealand, Wright *et al*^36^ evaluated MatCHAT as a codesigned e-screening and decision-support tool, while valued for data capture and resources, broader adoption was limited by translation needs and concerns about replacing face-to-face interactions.

#### RMC and shared decision-making

RMC and SDM are also important. In Tanzania, Sequeira D’mello *et al*^52^ developed the RMC-T to assess and improve women’s experiences, identifying communication gaps, provider biases and mistreatment concerns that were addressed via codesigned educational materials and staff training; reported outcomes included improved empathy, reduced mistreatment and higher satisfaction among providers. In the Netherlands, Murugesu *et al*^53^ codeveloped a conversation aid and training to strengthen SDM, reporting improved health literacy, confidence and communication skills. In the UK, Whybrow *et al*^34^ implemented an SDM intervention for pregnant women with chronic hypertension, codesigned decision aids, infographics and staff training, reporting reduced decisional conflict (mean reduction of 42%) and increased willingness to take antihypertensive medication. Acceptability, appropriateness and feasibility were judged to be high, with recommendations for multilingual resources and interpreter access to support equity in healthcare.

### Codesign processes and implementation determinants

Codesign shows clear promise in maternity care and service redesign, but its effects are sometimes shaped by on-the-ground reality. Ethnographic work on adapting intrapartum guidelines in Tanzania shows how power asymmetries surface throughout cocreation (who sets agendas, whose evidence ‘counts,’ who speaks in mixed-cadre rooms), often tilting participation towards consultation rather than SDM unless processes explicitly redistribute voice (eg, facility-led participant selection, shared facilitation and early, transparent planning).^40^

Operational determinants are equally influential. In a midwife-led quality-improvement cocreation, participants repeatedly identified workload, limited space, high patient volumes, staff turnover and uneven skills mix as reasons that seemingly ‘simple’ evidence-based practices (continuous support, alternative birth positions, perineal protection) stall without protected time, champions and train-the-trainer structures.^54^ These constraints also explain why teams gravitate towards what can be delivered within the existing infrastructure and routines.

The sociocultural context further shapes this uptake. Women in rural Tanzania described fragmented, one-way communication, normalised mistreatment and exclusion from decisions; expectations for empathic, respectful care were often low and gatekeeping around companionship remained common because of norms and space constraints.^55^ Codesigned improvements that elevate women’s voice, strengthen respectful communication and address structural barriers (eg, space for companions) are therefore preconditions for meaningful participation, not ‘nice-to-haves.’

Countervailing facilitators have been well documented. In Australia, a codesigned, community-governed ‘Birthing on Country’ service that integrates continuity of midwifery carers, a strengthened First Nations workforce and culturally safe wrap-around supports improved core outcomes (greater antenatal engagement, lower preterm birth and higher exclusive breastfeeding) relative to standard care, demonstrating how multiagency partnership and community governance can translate cocreation into durable system change.^56^

Finally, iterative feedback loops operationalise learning. Experience-based co-design in a UK maternity pathway used interviews with surface ‘touchpoints,’ separate and joint workshops to negotiate priorities, and staged redesign with visible pathway changes (‘you said-we did’), illustrating how reflexive monitoring sustains fidelity to user intent while enabling adaptation.^27^

### Risk of bias and quality assessment

The quality of cohort and case–control studies was assessed using the NOS (figure 2).^22^ The quality of each RCT was assessed using the Cochrane RoB2 tool (figure 3).^23^ For all other non-randomised studies, we incorporated the ROBINS-E tool (figure 4) to examine potential biases.^24^ The risk of bias and quality assessment also employed a similar approach as described in the Data extraction section, with conflicts resolved through discussion.

**Figure 2:**
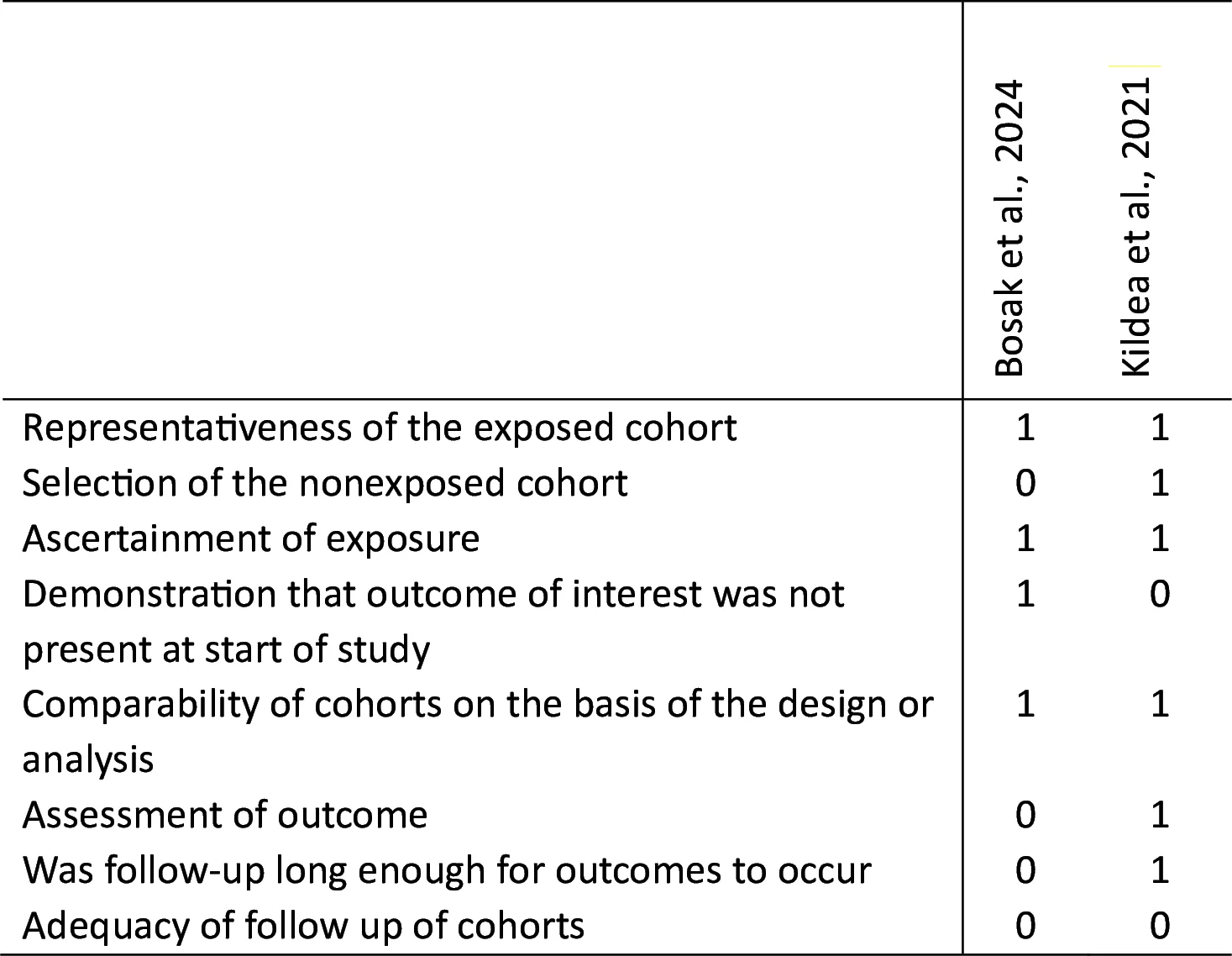
Newcastle-Ottawa Scale (NOS) for assessing the quality of the cohort studies (n=2).^22^

**Figure 3:**
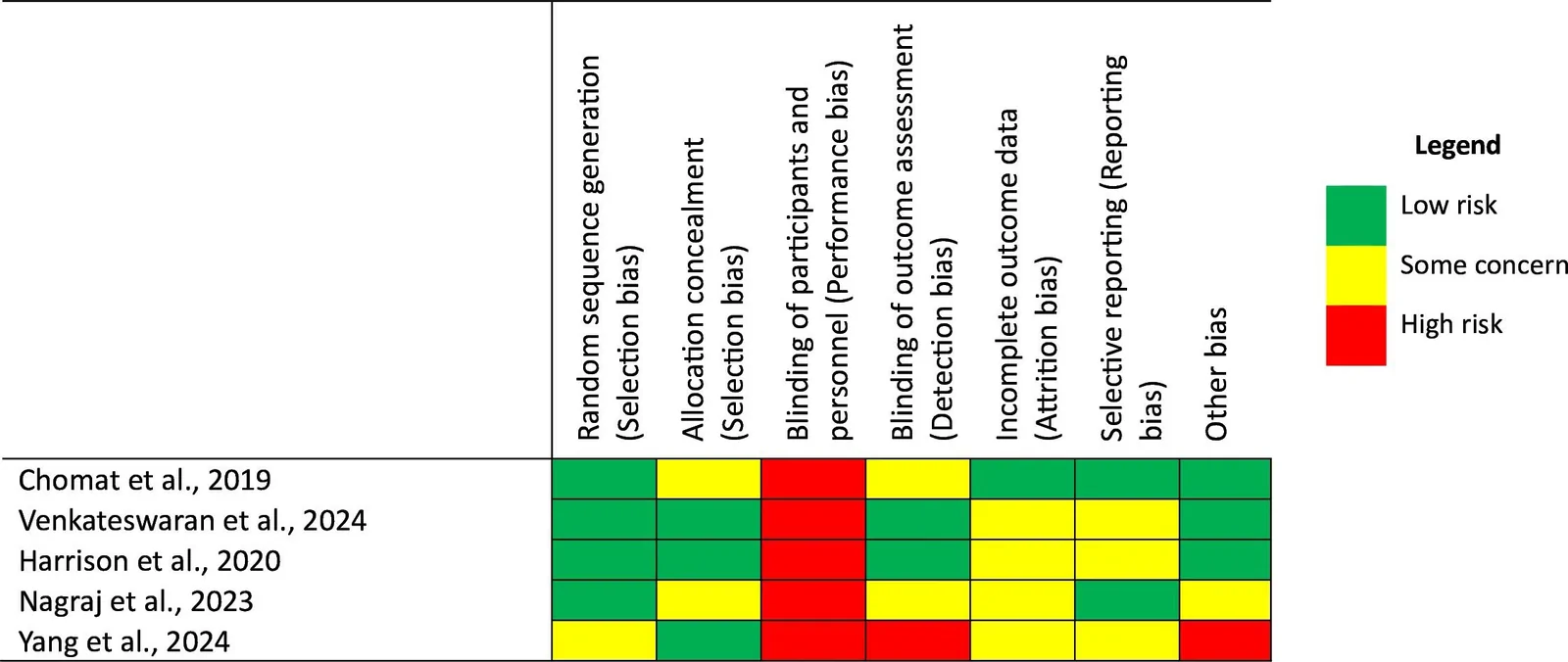
Cochrane risk-of-bias (RoB2) tool for the assessment of randomised controlled trials included in the systematic review (n=5).^23^

**Figure 4:**
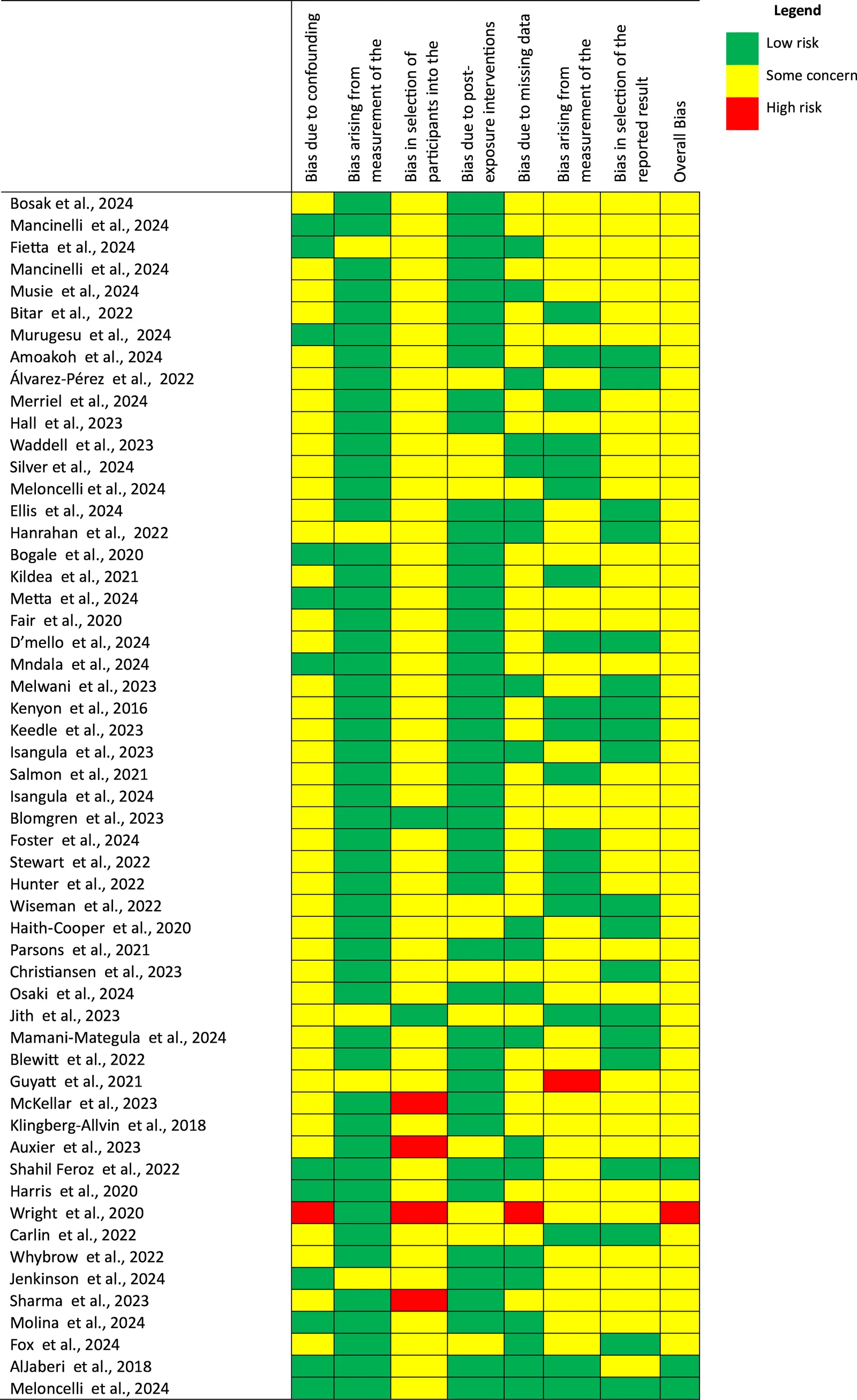
Assessment of the risk of bias in non-randomised studies via the Risk of Bias In Non-Randomised Studies - of Exposure (ROBINS E tool) (n=55*).*Including the two cohort studies which were also assessed using Newcastle-Ottawa Scale.^24^

## Discussion

The integration of cocreation and codesign methodologies into obstetric and maternal healthcare has demonstrated transformative potential, fostering culturally relevant, patient-centred interventions that address systemic challenges in care quality, accessibility and equity. This systematic review synthesises evidence from 60 studies across diverse settings, revealing three core themes: (1) the adaptability of codesign to address context-specific barriers, (2) its role in enhancing stakeholder engagement and trust and (3) its capacity to drive innovation in digital and mental health interventions. Below, we contextualise these findings within the broader literature, outline their unique implications for maternal healthcare and propose actionable directions for future research and practice.

### Key contributions and alignment with the literature

Our findings align with codesign applications in other health domains, where stakeholder collaboration improves intervention relevance and patient satisfaction.^3 4^ For example, digital tools such as SMARThealth Pregnancy^31^ and eRegMat^50^ mirror successes in chronic disease management, where UCD enhances adherence and usability. Similarly, improved provider–client relationships through codesigned training programmes^25 26 53^ resonate with advancements in palliative care, where communication tools strengthen therapeutic alliances.^10^ However, maternal healthcare distinctively prioritises cultural congruence, as seen in interventions such as women’s circles^30^ and Birthing on Country models,^56^ which integrate traditional practices to bridge gaps in care access, which is less pronounced in standardised chronic disease care frameworks. This emphasis on cultural safety highlights the need for hyper-localised approaches to maternal health, where generational and gender-based inequities disproportionately affect outcomes.^57^ Consistent with the literature, our synthesis indicates that the primary mechanism of codesign in maternity care is relational and power attending; it builds trust, shared understanding and contextual fit, thereby supporting communication, SDM, cultural safety and implementation, rather than the development of novel pharmacologic or surgical interventions.

### Challenges and unique considerations

While the benefits of codesign are evident, implementation barriers, hierarchical power dynamics, resource constraints and stakeholder fatigue echo the challenges observed in mental health and digital innovation studies.^35 36 58^ Maternal healthcare faces added complexities, such as a dual focus on immediate clinical outcomes and long-term and generational impacts. For example, gestational diabetes prevention programmes^59^ and anaemia management strategies^28^ not only improve maternal health but also mitigate intergenerational risks, necessitating sustained engagement, which is uncommon in acute care contexts. Furthermore, the integration of digital solutions in low-resource settings^35 49^ highlights the tension between technological feasibility and cultural acceptability, which requires iterative adaptation that is not present in high-resource settings.

### Implications for policy and practice

To maximise the impact, policymakers should prioritise three key areas: (1) scalable community-led models (programmes such as Tanzania’s RMC-T^52^ and Malawi’s antenatal case notes^60^ demonstrate that locally adapted tools, developed with healthcare workers and patients, improve care adherence and reduce mistreatment); (2) digital equity (investments in low-tech platforms (eg, SMS-based systems^50^) and telehealth infrastructure can bridge gaps in rural and underserved regions, provided they are codesigned with end users to address literacy and access barriers) and (3) workforce training (midwifery education frameworks,^61^ educational interventions for continuity of care mode,^62^ and SDM aids^34 53^ warranting the need for codesigned training programmes that simultaneously enhance provider empathy and technical skills). Furthermore, policymakers should continue to invest in cocreation/codesign capacity and its transparent reporting, especially in digital and implementation contexts, to maximise the likelihood that policies and services are implementable, context-fit and adopted at scale.

### Future research directions

Future studies should prioritise the long-term efficacy of these treatment options. Few studies have assessed the durability of the codesigned interventions. Longitudinal evaluations are critical for understanding sustainability. Another area of focus is digital mental health; scalable, low-cost tools require testing in diverse populations to address postpartum depression and anxiety. Finally, using intersectional approaches, research must explore how codesign can address overlapping inequities to ensure inclusive solutions.

### Limitations

This systematic review has several limitations that must be acknowledged. First, the inclusion criteria were restricted to full-text articles published in English from 2014 onward, which may have introduced language and publication bias by excluding relevant studies published in other languages or outside the selected timeframe. Second, the review encompassed a wide variety of study designs, including RCTs, pilot studies, mixed methods and qualitative approaches, which, while reflecting the multifaceted nature of cocreation and co-design in maternal health, resulted in considerable heterogeneity in the findings. This heterogeneity precluded the possibility of conducting a meta-analysis and limited the ability to draw firm conclusions regarding the effectiveness of the interventions studied. Third, our search strategy prioritised studies that documented codesign/cocreation activities and, given heterogeneous reporting, did not systematically classify primary intent (ie, codesign to develop an intervention vs evaluation of a codesigned intervention), which may limit interpretability across aims, despite our narrative grouping by stated intents.

Furthermore, the exclusion of grey literature might have omitted additional evidence that could provide further insight into the application and impact of codesign methodologies in diverse settings. The inherent complexity of codesign interventions, characterised by their multifaceted processes and context-specific adaptations, further complicates the attribution of observed outcomes solely to the cocreation process. External factors, such as resource availability and local contextual differences, may have confounded the results, making it difficult to isolate the specific contribution of codesign to improved maternal health outcomes.

Another notable limitation is the scarcity of long-term follow-up data in many of the included studies, which restricted the evaluation of the sustainability and enduring impact of codesigned interventions on maternal health outcomes. Finally, the lack of standardised definitions and evaluation frameworks for cocreation and codesign across studies poses challenges for comparability and generalisability, emphasising the need for future research to adopt more uniform reporting and assessment tools to address this gap. Despite these limitations, this review provides valuable insights into the potential benefits and challenges of integrating codesign methodologies into obstetric and maternal healthcare.

## Conclusion

By focusing on stakeholder voices, codesign methodologies offer a pathway to equitable and innovative maternal healthcare. Their success hinges on contextual adaptability, sustained community engagement and policy support, principles that must guide future research and implementation to achieve global maternal health equity.

## Supplementary material

10.1136/bmjqs-2025-019030 online supplemental file 1

10.1136/bmjqs-2025-019030 online supplemental file 2

## Data Availability

All data relevant to the study are included in the article or uploaded as supplementary information.
