# Supplementary material for: Cocreation and codesign in maternal health: processes, outcomes and implementation challenges
Source: BMJ Qual Saf. 2026 Feb 20;35(10):e019030. doi: 10.1136/bmjqs-2025-019030 (PMC13638332; doi:10.1136/bmjqs-2025-019030)
Supplement: Supplementary file 1 [file bmjqs-35-10-s001.docx]

# Full Search Strategies by Database

## PubMed

("codesign"[Title/Abstract] OR "co-design"[Title/Abstract] OR "cocreation"[Title/Abstract] OR "co-creation"[Title/Abstract] OR "participatory design"[Title/Abstract] OR
"user-centered design"[Title/Abstract] OR "user-centred design"[Title/Abstract] OR "co-production"[Title/Abstract] OR "coproduction"[Title/Abstract] OR
"stakeholder engagement"[Title/Abstract] OR "stakeholder involvement"[Title/Abstract])
AND
("obstetric care"[Title/Abstract] OR "maternal health"[Title/Abstract] OR "childbirth"[Title/Abstract] OR "intrapartum care"[Title/Abstract] OR
"pregnancy"[Title/Abstract] OR "maternity services"[Title/Abstract] OR "perinatal care"[Title/Abstract])
AND
("2014/01/01"[Date - Publication] : "2025/01/01"[Date - Publication])

## MEDLINE (via OVID)

1. exp Patient Participation/
2. (codesign* or co-design* or cocreation* or co-creation* or participatory design* or user-centred design* or user-centered design* or co-production* or coproduction* or stakeholder* engagement or stakeholder* involv*).tw.
3. 1 OR 2
4. exp Maternal Health Services/ OR exp Pregnancy/ OR exp Obstetric Delivery/
5. (obstetric care or maternal health or childbirth or intrapartum care or pregnancy or maternity services or perinatal care).tw.
6. 4 OR 5
7. 3 AND 6
8. limit 7 to (english language and humans and yr="2014 -Current")

## Embase

('codesign'/exp OR 'co-design':ti,ab OR 'cocreation':ti,ab OR 'co-creation':ti,ab OR 'participatory design':ti,ab OR 'user-centered design':ti,ab OR 'user-centred design':ti,ab OR
'co-production':ti,ab OR 'coproduction':ti,ab OR 'stakeholder engagement':ti,ab OR 'stakeholder involvement':ti,ab)
AND
('obstetric care'/exp OR 'maternal health'/exp OR 'pregnancy'/exp OR 'childbirth':ti,ab OR 'intrapartum care':ti,ab OR 'perinatal care':ti,ab OR 'maternity services':ti,ab)
AND
[2014-2025]/py AND [english]/lim AND [human]/lim

## ScienceDirect

TITLE-ABSTR-KEY(codesign* OR co-design* OR cocreation* OR co-creation* OR participatory design* OR user-centered design* OR user-centred design* OR
coproduction* OR co-production* OR stakeholder* engagement OR stakeholder* involv*)
AND
TITLE-ABSTR-KEY(obstetric care OR maternal health OR childbirth OR intrapartum care OR pregnancy OR perinatal care OR maternity services)
AND
PUBYEAR > 2013 AND PUBYEAR < 2025 AND LANGUAGE(english)

## Cochrane Library

(codesign*:ti,ab OR co-design*:ti,ab OR cocreation*:ti,ab OR co-creation*:ti,ab OR participatory design*:ti,ab OR user-centred design*:ti,ab OR user-centered design*:ti,ab OR
co-production*:ti,ab OR coproduction*:ti,ab OR stakeholder engagement:ti,ab OR stakeholder involvement:ti,ab)
AND
(obstetric care:ti,ab OR maternal health:ti,ab OR childbirth:ti,ab OR intrapartum care:ti,ab OR pregnancy:ti,ab OR perinatal care:ti,ab OR maternity services:ti,ab)
AND
(date >= 2014 and date <= 2025)
