# Supplementary material for: Cocreation and codesign in maternal health: processes, outcomes and implementation challenges
Source: BMJ Qual Saf. 2026 Feb 20;35(10):e019030. doi: 10.1136/bmjqs-2025-019030 (PMC13638332; doi:10.1136/bmjqs-2025-019030)
Supplement: Supplementary file 2 [file bmjqs-35-10-s002.docx]

| **S No.** | **Title** | **Author**  **Year**  **Country** | **Aim of the Study** | **Methodology** | **Co-approach and Framework** | **Participants** | **Intervention** | **Outcome measure** | **Conclusion** |
| --- | --- | --- | --- | --- | --- | --- | --- | --- | --- |
| 1 | Interventions co-designed by healthcare providers and clients for improving therapeutic relationships in maternal and child healthcare: a pilot study using human centered design in rural Tanzania | Isangula et al.  2023  Tanzania [25] | To jointly create a prototype intervention package for enhancing nurse‑client relationships using human centered design (HCD) approach | A five-step Human Centered Design approach was briefly used to co-create interventions for strengthening nurse-client relationships | Co-creation and co-design | A transdisciplinary team of purposively sampled MCH nurses and midwives, clients, administrators, and other relevant stakeholders (30 members) gathered to define the challenges based on discovery findings and design an intervention package | Refinement meetings resulted in a final prototype including four interventions:  Promotion of patient‑centered care  Awards and recognition for nurses  Strengthening complaints mechanisms  Disciplinary measures for abusive nurses and clients | Nurse factors contributing to poor nurse-client relationships include poor reception, lack of care, communication issues, negative attitudes, low service quality, work unhappiness, and unstable mental health. Client factors include lateness, rule disregard, negative attitudes, poor communication, lack of education, poverty, dissatisfaction with care, faith in alternative healers, and unstable mental health. | HCD approach provides a novel entry point for providers and clients to examine the problems and design interventions for strengthening their therapeutic relationships in MCH care. |
| 2 | Nurses’ and clients’ perspectives after engagement in the co-designing of solutions to improve provider-client relationships in maternal and child healthcare: a human centered design study in rural Tanzania | Isangula et al.  2024  Tanzania [26] | To explore participants’ view points following their participation in the co-design phase of a HCD study, designed to devise solutions for enhancing provider-client relationships in the context of MCH care in rural Tanzania | A qualitative descriptive design was used - Human-centered design (HCD) approach | Co-design | A transdisciplinary team of purposefully selected MCH nurses and midwives, clients and MCH stakeholders including regional and municipal MCH administrators, and local non-governmental organizations | Three main themes were developed from the analysis, encompassing key learnings from engagement in the co-design process, the potential benefits of co-designing interventions, and co-designing as a tool for behavior change and personal commitment |  | End-users’ perspectives after engagement in the co-design process suggest it provides a novel entry point for strengthening provider-client relationships and addressing other health sector challenges |
| 3 | Improving the care pathway for women who request Caesarean section: an experience-based co-design study | Kenyon et al.  2016  England [27] | To improve services and outcomes for patients who request Caesarean section within five years | Experience-based co-design methodology uses service user and clinicians’ experiences collected using qualitative methods to jointly re-design services. | Experience-based-Co-design method | The core team consisted of the researcher with a clinical midwifery background, sociologist and methodological specialist, research fellow, obstetrician and service user/mother offering a range of perspectives  on the project | Changes to the pathway include providing written information on "The way your baby may be born" at the 16-week appointment, followed by a discussion on birth options. If a woman requests a Caesarean, she's referred to appropriate professionals for support and information. A referral to a consultant obstetrician is made at 20 weeks, with a decision by 28 weeks. Recording this in the notes reduces repeated challenges, while final consent and timing for the Caesarean remain as recommended. |  | This has resulted in changes to the pathway agreed by a co-design process and which are acceptable to both health care professionals and women |
| 4 | Using an experience‑based codesign approach to develop strategies for implementing an intravenous iron intervention to treat moderate and severe anemia in pregnancy in Malawi | Mamani‑Mategula et al.  2024  Malawi [28] | To outline the application of an experience-based co-design (EBCD) approach to-  Identify key touchpoints, influencing the barriers and facilitators to IV iron use to treat anemia in pregnancy in the primary healthcare system of Malawi  Develop mitigating strategies for the successful implementation of REVAMP-TT trial |  | Co-design  Experience‑based co‑design approach | Two co‑design workshops with end‑users (n = 20) and healthcare providers (n = 20) | The proposed strategies to mitigate the barriers for the successful implementation of the REVAMP TT trial included providing financial strategy, developing stake holder relationships, training and educating stakeholders, supporting clinicians, and engaging end‑users. |  | The experience-based co-design approach revealed barriers and facilitators to IV iron use, aiding the successful implementation of the REVAMP-TT trial. Engaging key informants and end users fostered collaboration, ownership, and shared real-world insights. |
| 5 | The role of local governments in the implementation of evidence-based nutrition interventions in Kerala | Jith et al.  2023  India [29] | To study the involvement and influence of local participatory governments consisting of Panchayat Raj institutions (PRIs) in implementing evidence-based interventions for eliminating maternal and child undernutrition in the state of Kerala, India | Qualitative methods were used to meet the research objectives | Co-creation | PRIs have created spaces of co-creation and collaboration where elected representatives, community- based organizations (CBOs), religious organizations and civil society organizations (CSOs) can come together and participate in maternal and child health interventions | PRIs supported nutrition interventions by mobilizing resources, monitoring, engaging communities, and fostering participation through formal and informal platforms |  | Results illustrate complex linkages PRIs have within the health system and how these linkages help in the implementation of interventions |
| 6 | Women’s circles as a culturally safe psychosocial intervention in Guatemalan indigenous communities: a community-led pilot randomized trial | Chomat et al.  2019  Guatemala [30] | To assess co-designed Women’s Circles’ in terms of accept ability, feasibility and proof-of-concept in preparation for a future definitive trial. | Parallel group pilot randomized study | Co‐design | The participatory research approach involved initial needs assessment and co‐design, co‐development, and co‐implementation of the intervention with the 16 circle leaders | The program incorporated individual and group activities using evidence-based methods like CBT, IPT, education, mindfulness, and vocational therapy to enhance livelihood skills and support small-scale income generation. | Primary outcomes  Maternal symptoms of depression and anxiety over the last month  Maternal wellbeing  Self‐efficacy  Mother’s engagement in early infant stimulation  Secondary outcomes  Acceptability was assessed in post‐intervention focus groups and in‐depth interviews  Intervention feasibility | The pilot showed promise for a future randomized trial, demonstrating feasibility and acceptability. Co-designed women's groups offer indigenous women a safe space to enhance wellbeing and functioning. |
| 7 | A Mobile Clinical Decision Support System for High-Risk Pregnant Women in Rural India (SMARThealth Pregnancy): Pilot Cluster Randomized Controlled Trial | Nagraj et al.  2023  India [31] | To co-design a theory-informed intervention for CHWs to screen, refer, and counsel pregnant women at high risk of future CVD in rural India and evaluate its feasibility and acceptability | The design and development of SMARThealth Pregnancy occurred in 3 phases, guided by the methodological principles of human-centered design (inspiration, ideation, and implementation) | Co-design | ASHAs and primary care doctors co-designed SMARThealth Pregnancy | SMARThealth Pregnancy integrated 15 behavior change techniques, featuring community awareness programs, targeted training for CHWs (including point-of-care tests), and mobile clinical decision support for home screening. |  | SMARThealth Pregnancy is an innovative home-based care model for high-risk pregnancies, with theory and co-design enhancing its acceptability and implementation. |
| 8 | Group-based pelvic floor muscle training for pregnant women: A randomized controlled feasibility study | Yang et al.  2024  China [32] | To test the feasibility and acceptability of a group-based pelvic floor muscle training for pregnant women in China and facilitate women's adherence to the pelvic floor muscle training program | A single-center, two-arm feasibility randomized controlled trial (RCT) - mixed methods process evaluation | Co-design | The patient and public have been invited as stake-holders during the development of the intervention. | Participants attended four face-to-face sessions led by the research midwife and were encouraged to perform PFMT twice daily at home. They received training booklets, a diary, and reminder stickers, along with automated reminders and audio in the WeChat group to guide exercises. |  | The study showed promising results concerning the acceptability and feasibility of the intervention, which were well perceived by both pregnant women and the midwife. |
| 9 | Development of a targeted client communication intervention to women using an electronic maternal and child health registry: a qualitative study | Bogale et al.  2020  Palestine [33] | To develop interview guides to explore women’s perceptions of antenatal care (ANC), with a focus on high-risk pregnancy conditions (anemia, hypertensive disorders in pregnancy, gestational diabetes mellitus, and fetal growth restriction), and untimely ANC attendance, issues predefined by a national expert panel as being of high interest |  | Co-design | We performed 18 in-depth interviews with women, and eight with healthcare providers in public primary healthcare clinics in the West Bank and Gaza | To increase the utilization of ANC and bridge the identified gaps, we iteratively composed actionable text messages with users, using recommended message framing models. We developed algorithms to trigger tailored text messages with higher intensity for women with a higher risk profile documented in the electronic health registry |  | We developed an optimized TCC intervention underpinned by behavior change theory and concepts, and co-designed with users following an iterative process. The electronic maternal and child health registry can serve as a unique platform for TCC interventions using text messages |
| 10 | Implementation of a novel shared decision-making intervention in women with chronic hypertension in pregnancy: multiple-site multiple-method investigation | Whybrow et al.  2022  United Kingdom [34] | To codesign, implement and evaluate a novel shared decision-making (SDM) intervention for use with pregnant women with chronic hypertension | The Chronic Hypertension in pregnAncy iMPlementatION study (CHAMPION) is a multiple-method investigation into the codesign and implementation of a novel SDM intervention for women with chronic hypertension in pregnancy. | Co-design | Pregnant women with chronic hypertension and their principal healthcare professionals (obstetricians, midwives, and physicians) | Development of a novel shared decision-making (SDM) intervention for use with pregnant women with chronic hypertension. | Primary: Decisional conflict scale, medication intention survey and women’s acceptability.  Secondary: Healthcare professionals’ acceptability and the barriers and facilitators to SDM implementation with pregnant women with chronic hypertension | A novel codesigned SDM intervention reduced decisional conflict and increased women’s intention to take antihypertensive agents during pregnancy. This intervention could be adopted into practice for women making pregnancy decisions where there is uncertainty around the medication management option. |
| 11 | YourTime: The development and pilot of a perinatal mental wellbeing digital tool using a co-design approach | McKellar et al.  2023 [35] | To build on the original research to co-design and test a prototype digital perinatal mental health tool to enable women to track their mental wellbeing over time, alert them to changes and assist them to recognize early signs of deteriorating mental wellbeing.  To promote mental health by incorporatingwellbeing prompts based on the ‘five-ways to wellbeing’. | A sequential mixed methods approach undertaken in two stages 1) co-design workshops; 2) fit for purpose pilot undertaken late 2019 through to 2020. | Co-design | Mothers, midwives, design researchers and students, participated in co-designing a digital tool and prototype application | The perinatal mental health digital tool – YourTime app  Mediation (paced breathing)  Breathing (relaxed breathing and deep breathing)  Journaling (keep a record of how you were feeling)  Affirmations (positive written statements)  Network (being able to add contacts) |  | The YourTime app shows promise in providing a digital resource for women to self-assess and monitor their mental wellbeing over time and may promote selfcare and help-seeking behaviour. Working with the end users of the digital resource is critical to better understand the needs and contexts in which these resources will be used. |
| 12 | Perinatal e-screening and clinical decision support: the Maternity Case-finding Help Assessment Tool (MatCHAT) | Wright et al.  2020  New Zealand [36] | To assess the acceptability and feasibility of the Maternity Case-finding Help Assessment Tool (MatCHAT), a tool designed to provide e-screening and clinical decision support for depression, anxiety, cigarette smoking, use of alcohol or illicit substances, and family violence among pre- and post-partum women under the care of midwives. | The study used co-design and a qualitative  approach | Co-design | Extensive consultation with  midwives, physicians and hospital administration | Development of the Maternity Case-finding Help Assessment Tool  (MatCHAT) |  | MatCHAT shows promise for improving e-screening and decision support in maternity care, but contextual barriers limited its use. These findings can inform policy and future efforts to enhance early detection of mental health, substance use, and family violence issues. |
| 13 | Healthy weight services in England before, during and after pregnancy: a mixed methods approach | Fair et al.  2020  England [37] | To explore current service provision of maternal healthy weight services in England through a survey of LMS representatives and to determine the perspectives of key stakeholders on variation in service provision and related barriers and facilitators locally | A mixed methods approach was undertaken | Co-creation and co-design | Stakeholders included providers and commissioners of maternity, public health, sexual health, contraceptive, health visiting services and service users  Clear communication with service users to ensure ap propriate services are commissioned was considered key to ensure access | Using an online Survey Monkey questionnaire, to establish the various range and type of services available to support women in achieving a healthy weight and lifestyle prior to pregnancy, during pregnancy or up to 1 year postpartum |  | It's crucial that all healthcare workers are aware of relevant services. More maternity healthy weight services, focusing on physical activity, are needed, particularly for those with a raised BMI, both pre-pregnancy and postnatally. Robust evaluation is required to ensure these services are effective. |
| 14 | Development and evaluation of an online education module to improve clinician knowledge and management of eating disorders in the peripartum: a pre-post survey study | Ellis et al.  2024  Australia [38] | To develop an online educational module for clinicians and support workers who work with perinatal individuals who may experience ED, have disordered eating, or have a history of ED and are vulnerable to relapse | The study was a pre-post design using online surveys to evaluate changes in knowledge of eating disorders in the perinatal period after completing an online education module. | Co-design | The team included peer workers, midwives, medical specialists, nursing staff, and allied health staff who work in maternity and perinatal mental health services, and screen and/or manage perinatal individuals who may experience an eating disorder | Online education module |  | Findings indicate that the ED online education module is an engaging and easy-to-use tool for improving the knowledge and skills of the healthcare workforce, thereby improving patient care and health outcomes. |
| 15 | Implementation of the ‘Kimberley Mum’s Mood Scale’ across primary health care services in the Kimberley region of Western Australia: A mixed methods assessment | Carlin et al.  2022  Australia [39] | To assesses the implementation process and outcomes of the KMMS across the Kimberley region using these modified domains of the Dynamic Sustainability Framework. | This study uses a mixed methods approach to explore real-world outcomes of KMMS implementation in the Kimberley using the Dynamic Sustainability Framework | Co-design | Co-designed with Aboriginal women and healthcare professionals to improve culturally appropriate screening practices for perinatal depression and anxiety | Development and implementation of the Kimberley Mum’s Mood Scale (KMMS) |  | This study demonstrates that innovation in perinatal depression and anxiety screening for Aboriginal women is possible and can be implemented into routine clinical care with the support of a sustained multi-year investment and strong partnerships. |
| 16 | The Dilemmas and Opportunities of Co‐Creating Health Interventions to Fit Local Contexts: An Ethnographic Study on the Adaptation of Clinical Guidelines in Tanzania | Osaki et al.  2024  Tanzania [40] | To share the dilemmas of engaging healthcare providers in co‐creating locally adapted clinical guidelines for maternity facilities in Dar es Salaam, Tanzania, and strategies used to address them | An ethnographic approach was adopted to study the PartoMa guideline co‐creation process | Co-creation | Stakeholders included frontline health providers and healthcare managers from five maternity units, researchers, members from the regional and district health management teams and maternal health experts | Co‐creating locally adapted clinical guidelines |  | Key challenges in the co-creation process included contextual variation, differing knowledge systems, and power dynamics. However, reflexivity, open dialogue, and early engagement improved the process. Co-creating locally adapted guidelines with frontline workers and experts is crucial for feasibility and safety. |
| 17 | Social singing, culture and health: interdisciplinary insights from the CHIME project for perinatal mental health in The Gambia | Stewart et al.  2020  Gambia [41] | To develop an understanding, both of how mental distress during the perinatal period is understood and experienced in the Gambian context, as well as the existing musical practices associated with the perinatal period, and attitudes towards them | The development of the intervention was informed by principles of co-design and participatory ethnography | Co-creation and co-design | Group discussions with pregnant women, midwives, community birth companions, Kanyelengs and griots (hereditary musicians) |  |  | Designing health interventions based on cultural practices requires partnerships across disciplines with broad representation at the community, civic, and government levels. |
| 18 | Social Interaction Needs and Entertainment Approaches to Pregnancy Well-Being in mHealth Technology Design for Low-Income Transmigrant Women: Qualitative Codesign Study | AlJaberi et al.  2018  United States of America [42] | To reveal the needs and gaps in mHealth interventions for pregnant immigrant women not yet realized in this field | Qualitative participatory action research approach with codesign | Co-design |  | Development of mHealth pregnancy management interventions |  | The study explored how pregnant immigrant women share health information online, including their preferred sharing methods and media. It also highlighted gaps in current health technologies that limit their social sharing options. |
| 19 | Qualitative evaluation of a package of implementation strategies codesigned to support the introduction of multiple micronutrient supplementation (MMS) for pregnant women in Bamako, Mali | Fox et al.  2024  Mali [43] | To inform ANC delivery strategies to increase adherence to antenatal MMS, Jhpiego in partnership with the Center for Vaccine Development in Mali (CVD‐Mali), Mali Ministry of Health and the Johns Hopkins Bloomberg School of Public Health (BSPH)—Center for Human Nutrition conducted implementation research to compare adherence to and acceptability of three different delivery approaches to supplementation in pregnancy. | Qualitative and mixed methods study | Co-design | Midwives, women who were pregnant and/or mothers of young children, graphic designers, representatives of Jhpiego, the Ministry of Health, a local university and a local youth health advocacy group participated in a 2 day workshop to codesign a local name, label and packaging for the MMS product and a set of counselling messages to encourage women to take MMS daily | Development of antenatal care MMS packaging and counselling materials aimed at optimizing delivery and uptake of and adherence to MMS |  | The evaluation shows MMS is well accepted in this Malian community. Shifting from IFA to MMS will require training, resources, and support for ANC providers. The materials tested can help launch community-based strategies to boost adherence. Since concerns and misinformation about pregnancy supplements are common, the brief codesign process used here could guide other countries in adapting MMS rollout to fit their local context and health systems. |
| 20 | Understanding the Needs of a Mobile Phone–Based Telemonitoring Program for Pregnant Women at High Risk for Pre-Eclampsia: Interpretive Qualitative Description Study | Feroz et al.  2022  Pakistan [44] | To explore the needs of TM for pregnant women at high risk for pre-eclampsia in Karachi, Pakistan  To inform a potential future feasibility trial of a mobile phone–based TM program. | An interpretive qualitative description approach | Co-design | Pregnant women and key informants, including clinicians; nurses; maternal, neonatal, and child health specialists; and digital health experts | Development of a mobile based telemonitoring program for pregnant women with high risk for pre-eclampsia |  | Our findings support the feasibility of a mobile TM program for high-risk pregnant women and offer insights to guide future designs aimed at reducing pre-eclampsia-related mortality in LMICs. |
| 21 | Partnering with the woman who declines recommended maternity care: Development of a statewide guideline in Queensland, Australia | Jenkinson et al.  2024  Australia [45] | To describe the process and outcomes of co-designing resources to support partnership between the woman who declines recommended maternity care and the clinicians and health services who provide her care. | A participatory co-design process using Improvement Science's Plan-Do-Study-Act cycles. | Co-design | Consumer representatives, obstetricians, midwives, maternal fetal medicine specialists, neonatologists, health service executives, and legal and ethics experts | Development of the Guideline: Partnering with the woman who declines recommended maternity care, and associated resources. |  | Queensland is the first Australian jurisdiction, and perhaps internationally, to formally establish this kind of guidance in clinical practice. Such guidance is identified as an enabler of choice in the national Australian strategy *Woman-centered care: Strategic directions for Australian maternity services*. |
| 22 | Leveraging human centered design to enhance clinician communication during pregnancy care: Overcoming language barriers with Spanish-speaking patients | Molina et al.  2024  United States of America [46] | To develop a clinician communication tool to enhance patient trust in pregnancy care clinicians, especially among minoritized populations who face language and cultural barriers, using human-centered design | We applied an equity-driven HCD process to understand the problem, co-design a tool, and prepare for testing. | Co-design | We centered end-user experiences, including clinicians, Spanish-speaking patients, and Spanish-speaking cultural brokers, in our design process through multiple feedback sessions and modalities. | Development of a communication tool to enhance clinical communication during pregnancy care |  | Our HCD process highlights opportunities for meaningfully and productively engaging with patients across language differences in developing patient-centered innovations. The unique partnership between designers, researchers, and patients requires multilingual communication, mutual respect, and trust to embark on a shared design journey. |
| 23 | Development, acceptability and feasibility of a community-based intervention to increase timely initiation of antenatal care in an area of high ethnic diversity and low socio-economic status in the UK | Sharma et al.  2023  United Kingdom [47] | To develop and evaluate the acceptability and feasibility of a co-produced community-based intervention to increase uptake of antenatal care in an area with high ethnic diversity and low socio-economic status. | In this study, we employed a mixed-methods design comprising quantitative and qualitative methods to assess the acceptability and feasibility of the community-based intervention. | Co-production | 20 local service users and 14 stakeholders | Development of a community-based intervention to increase early initiation of antenatal care based on Diffusion of Innovations theory |  | The community-based intervention, coproduced with women and maternity care stakeholders, was positively evaluated, and offered an innovative and promising approach to engage and educate women about the timely initiation of antenatal care in an ethnically diverse and socio-economically deprived community. |
| 24 | Co-designing a nature-based intervention to promote postnatal mental health for mothers and their infants: a complex intervention development study in England | Hall et al.  2023  England [48] | To plan and design a nature- based intervention to promote postnatal mental health using a person- based and co- design approach | Evidence- based, theory- based, person- based and co- design approaches informed the planning (stage 1) and design (stage 2) of the intervention | Co-design | The team includes experts in psychiatry, perinatal research, intervention development, qualitative methods, nature-based practice, perinatal mental health, community advocacy, and mothers with lived experience. | Guiding principles were refined during intervention development. Activities led by a nature-based practitioner include:  Sensory meditation.  Seated mindfulness.  Slow, guided walks observing nature.  Weekly themed nature-based activities aligned with pathways to nature connectedness.  Consultation workshops. | Improve maternal mental health and infant wellbeing  Improve overall maternal mood  Reduce overall maternal anxiety  Improve maternal and infant sleep and maternal fatigue | This article provides an example of how in- depth qualitative research and co- design processes can be applied to plan and design a nature- based intervention for mothers and infants, to promote postnatal mental health. Our research activities determined that the needs of our population were optimally met by tailoring existing nature- based interventions |
| 25 | A Digital Behavioral Activation Intervention (JuNEX) for Pregnant Women With Subclinical Depression Symptoms: Explorative Co-Design Study | Mancinelli et al.  2024  Italy [49] | To exploratorily investigate and compare the feasibility of the internet-based self-help guided versus unguided version of the Brief Behavioral Activation Treatment for Depression-Revised, an empirically supported in-person behavioral activation protocol, targeting pregnant women with subclinical depression symptoms | Explorative Co-Design Study | Co-design | Pregnant women | The Moodle platform delivered the intervention via 6 core and 3 optional e-learning modules. In the guided group, women had weekly chats with an assigned guide for homework support. The content included text, pictures, and videos. |  | Results show that replicating an in-person protocol digitally and using non-professional tools for self-help interventions made the approach unfeasible. However, pregnant women expressed interest in psychological support, especially through app-based digital psychosocial tools. |
| 26 | eRegMat – a digital registry for improved quality of antenatal care: a cluster-randomized trial in a rural area in Bangladesh | Venkateswaran et al.  2024  Bangladesh [50] | To evaluate the effect of an eRegistry compared to unshared digital client records in two rural sub-districts in Bangladesh. | A two-arm cluster-randomized controlled trial in primary care health facilities | Co-design | Co-designed with end users between 2016 and 2018 as per the protocol paper | The intervention was an eRegistry with three DHIs: health worker clinical decision support, feedback dashboards with action items, and targeted client communication via SMS | Primary - Timely attendance at eligible ANC visits according to national ANC guidelines, and hypertension screening and management  Secondary - Timely first ANC visit, high risk women successfully referred to a skilled provider for additional care, facility delivery, perinatal mortality and severe perinatal morbidities, and severe postpartum anemia | Intervention appeared to have an effect in reducing perinatal mortality and severe perinatal morbidities. However, the trial suffered several implementation challenges which precludes high certainty interpretations of the results. |
| 27 | Exploring Access as a Process of Adaptation in a Self-Monitoring Perinatal eHealth System: Mixed Methods Study from a Sociomaterial Perspective | Auxier et al.  2023  Finland [51] | To examine the processes occurring during the adapting of eHealth self-monitoring use, with a focus on the technology-pregnant user interactions | A mixed methods convergent evaluation design | Co-creation | Pregnant users | The ŌURA ring is a commercial smart ring that collects heart rate and variability, sleep, body temperature, respiratory rate, and physical activity data |  | A cocreation process between pregnant users and the eHealth system highlights access as key to personalization. Use varies based on individual preferences, habits, and values, shaped by both system mediation and user adaptation. |
| 28 | ‘I am happy to be listened to’: co-creation of a simple tool to measure women’s experiences of respectful maternity care in urban Tanzania | D’mello et al.  2024  Tanzania [52] | To co-create a routine RMC measurement tool (RMC-T) for congested maternity units in Dar es Salaam, Tanzania, and assess the RMC-T’s acceptability among women and healthcare stakeholders | A participatory, mixed-methods, human-centered approach based on an evidence-driven adaptive design for health care interventions (AHEAD) to co-create the tool | Co-creation | Stakeholders included postnatal women from the study facilities, front-line workers, midwives, obstetricians, health leaders from the health facilities, district and regional health officials, public health specialists, RMC advocates, and global health researchers | Development of a Respectful Maternity Care (RMC) tool | Measures include satisfaction, communication, mistreatment (e.g., abuse, neglect, discrimination), supportive care (e.g., food, mobility), birth companionship, pain relief, bed-sharing, and newborn respect. | The RMC-T is contextualized, validated, and acceptable for measuring women’s experiences of RMC. Routine use in facility-based quality improvement initiatives, along with targeted actions to address gaps, will advance rights-based RMC. Further validation and community-based studies are needed. |
| 29 | Co-creation of a health literate-sensitive training and conversation aid to support shared decision-making in maternity care | Murugesu et al.  2024  Netherlands [53] | To develop an SDM training for maternity care professionals and an SDM tool that would support HCPs and clients with varying HL levels in SDM | We combined elements of the Intervention Mapping protocol with User-centered design | Co-creation | The study included obstetricians, residents, nurses, midwives, maternity care assistants, lactation consultants, youth health specialists, women with a gestational age of 25 weeks or more, women who gave birth in the past year, and their partners. | Development of a conversation aid and health literate-sensitive training |  | The newly developed conversation aid and training have potential to support HCPs and clients in HL-sensitive SDM. However, they also perceived its use during consultations would be time consuming |
| 30 | Putting co-creation into practice: lessons learned from developing a midwife-led quality improvement intervention | Blomgren et al.  2023  Uganda [54] | To describe the co-creation process and explore the needs and determinants of a midwife-led quality improvement targeting evidence-based midwifery practices | A qualitative approach using the Consolidated Framework for Advancing Implementation Science (CFIR) | Co-creation | Midwives, professional association representatives, educators, policymakers, managers, doctors and women who recently gave birth and their birth companions | Peer-assisted learning, using existing structures, developing educational material, and building stakeholder relationships |  | This study details the co-creation process of a midwife-led QI intervention using the CFIR framework. It highlights the importance of involving stakeholders and placing midwives and end-users at the core, serving as a practical resource for advancing midwifery and patient-centered healthcare improvements. |
| 31 | Exploring women’s experiences of care during hospital childbirth in rural Tanzania: a qualitative study | Metta et al.  2024  Tanzania [55] | To explore childbirth experiences in Tanzania’s rural settings with the potential to integrate these experiences to co-design an intrapartum quality improvement intervention. | An exploratory qualitative study - initial phase of the co-design process for an intervention to intrapartum quality improvement known as ALERT | Co-design |  |  | Verbal mistreatment and threatening language for adverse birthing outcomes were common. Women appreciated physical or emotional support through human interaction. | The experiences of care described by women during childbirth varied from one woman to the other. Expectations towards empathic care seemed low, and the little interaction women had during labour and birth was therefore often appreciated and mistreatment normalized. |
| 32 | Effect of a Birthing on Country service redesign on maternal and neonatal health outcomes for First Nations Australians: a prospective, non-randomized, interventional trial | Kildea et al.  2021  Australia [56] | To assess and report the clinical effectiveness of the BiOC service on key maternal and infant health outcomes compared with that of standard care. | Prospective, non-randomized, interventional trial | Co-design | These services are co-designed with the local community and include continuity of midwifery carer, a First Nations workforce, partnerships between primary and tertiary services, cultural strengthening programmes, and wrap-around services to support pregnant women and their families | Birthing in Our Community (BiOC) service | Primary outcomes were antenatal care attendance, smoking after 20 weeks of gestation, preterm birth, and exclusive breastfeeding at discharge from hospital. | Our study found that the urban multi agency BiOC service redesign resulted in significantly improved maternal and infant outcomes, which were sustainable over time and are likely to affect individuals across the life course and intergenerationally |
| 33 | Co-designing Culturally Safe Indigenous Birth in High-Risk Obstetrics: Implementing Joyce’s Principle with Inuit and Cree Families and Their Medical Providers | Silver et al.  2024  Canada [57] | To implement co-designed cultural safety interventions in the context of maternal evacuation in collaboration with Inuit and Cree stakeholders | The work described in this article is the second phase of a long-term participatory study on culturally safe Indigenous birth | Co-design | We consulted 14 Inuit and Cree participants and 26 Indigenous and non-Indigenous perinatal providers (physicians, nurses, midwives, and community health workers) | Developing and Testing an Indigenous-Led Cultural Safety Training Program for Perinatal Staff  Increasing Family Presence and Support During Childbirth  Access to Traditional Country Foods  Supporting Inuit and Cree Perinatal Traditions |  | Culturally safe services are vital for supporting Indigenous families and reducing evacuation harms. A participatory research framework, guided by Joyce’s Principle, can enhance cultural safety in Indigenous childbirth evacuation by involving Indigenous stakeholders in co-design and implementation. |
| 34 | A Chatbot-Based Version of the World Health Organization–Validated Self-Help Plus Intervention for Stress Management: Co-Design and Usability Testing | Fietta et al.  2024 [58] | To adapt and co-design the World Health Organization’s Self-Help Plus intervention into a mobile health intervention for target groups (pregnant women and women diagnosed with breast cancer) | Holistic approach, merging diverse expertise and user-centric methodologies, | Co-design | Psychologists, communication experts, usability experts, pregnant women, women with breast cancer, gynecology clinicians and oncology clinicians | SH+ intervention for stress management through a mobile app guided by the ALBA chatbot |  | This research provides valuable insights for future studies aiming to enhance the usability, efficacy, and effectiveness of the app, suggesting the potential role of a chatbot-delivered Self-Help Plus intervention as a supportive tool for pregnant women and women with a breast cancer diagnosis. |
| 35 | Designing a behaviour change intervention using COM‐B and the Behaviour Change Wheel: Co‐designing the Healthy Gut Diet for preventing gestational diabetes | Meloncelli et al.  2024 [59] | To describe the development of the behavior, change dietary intervention, the Healthy Gut Diet | This qualitative study used co‐design to develop the behaviour change components of the Healthy Gut Diet intervention – COM B model of behavioral change | Co-design | The co‐design team was a partnership between study researchers, women with a lived experience of GDM (consumers) and workshop facilitators | The Healthy Gut Diet intervention used education, enablement, environmental restructuring, persuasion, and incentivization, incorporating 40 behavior change techniques across five delivery modes. |  | Using the Behaviour Change Wheel process in partnership with consumers resulted in a clearly described complex intervention targeting barriers and enablers of dietary behaviour change to improve the gut microbiota diversity in pregnant women |
| 36 | Developing comprehensive woman hand-held case notes to improve quality of antenatal care in low-income settings: participatory approach with maternal health stakeholders in Malawi | Mndala et al.  2024  Malawi [60] | To develop a compressive updated woman hand-held case notes tool (health passport book) which reflects WHO 2016 ANC guidelines in Malawi | A co-creative participatory approach in 3 workshops with key stakeholders to compare the current ANC tool contents to the WHO 2016 ANC guidelines | Co-creation | The team included obstetrics and gynecology specialists, registrars, clinical officers, nurse-midwives, district officers, safe motherhood coordinators, regulatory representatives, and patient representatives. | Development of the woman’s health passport book (ANC tool) |  | Our efforts reflect a pioneering attempt in Malawi to improve women’s hand-held case notes, which we know help in enhancing quality of care and improve overall women’s satisfaction with their healthcare system. |
| 37 | Strengthening global midwifery education to improve quality maternity care: Co-designing the World Health Organization Midwifery Assessment Tool for Education (MATE). | Hunter et al.  2022 [61] | To describe a three-phase co-designed project to develop a culturally appropriate and relevant education assessment tool, and report on pilot and field-testing phases | Three-phase co-design approach to develop, pilot and field-test an education assessment tool | Co-design | Experts in midwifery and nursing from Lithuania, Slovakia, Slovenia and Bulgaria.  The end-users were predominantly those providing midwifery and maternity care in the WHO European Region. | Development of a Midwifery Assessment Tool for Education (MATE) |  | Using a co-design approach has ensured that MATE is culturally relevant, accessible and appropriate. This initial evaluation indicates that MATE can facilitate in-country dialogue and support the strengthening of midwifery education in accordance with WHO aims. |
| 38 | Reflections on an educational intervention to encourage midwives to work in a continuity of care model –exploration and potential solutions | Harris et al.  2020 [62] | To explore barriers and facilitators for midwives working in a midwifery continuity of carer model, and to assess if an educational intervention could help address some of these barriers, designed to help achieve NHS England’s target of majority of women receiving midwifery continuity of carer by March 2021. |  | Co-design | The training package was co-designed by a panel of 27 staff with experience in providing or managing midwifery continuity models of care and/or education.  Experienced continuity midwives, service managers and midwifery educators. | Development of an educational training package |  | While this specific educational intervention appears to have been useful in addressing concerns with working in a continuity model, further work is needed to identify barriers to change. This will aid more local designed interventions |
| 39 | A Chatbot (Juno) Prototype to Deploy a Behavioral Activation Intervention to Pregnant Women: Qualitative Evaluation Using a Multiple Case Study | Mancinelli et al.  2024 [63] | To qualitatively evaluate pregnant women’s experiences with a chatbot (Juno) prototype designed to deploy a preventive behavioral activation intervention. | A multiple–case study design | Co-design |  | Development and Implementation of the Chatbot Juno | Depression symptoms were assessed using two self-report tools (including suicidal ideations), anxiety with the Generalized Anxiety Disorder-7, and stress with the Perceived Stress Scale-10. | The results highlighted both the strengths and limitations of the chatbot-based intervention, offering insights for refinement. Women emphasized the need to balance technology with human interaction, especially for reliable monitoring beyond preventive mental health contexts. |
| 40 | An Inclusive Framework for Collaboration between Midwives and Traditional Birth Attendants and Optimizing Maternal and Child Healthcare in Restricted Rural Communities in South Africa: Policy Considerations | Musie et al.  2024  South Africa [64] | To develop and validate an inclusive framework informed by the Donabedian structure-process-outcome (SPO) framework for collaboration between these healthcare professionals | The nominal group technique (NGT) was employed to enable engagement with key stakeholders across Africa | Co-creation | Key stakeholders as those with knowledge and years of experience in maternal healthcare services, researchers in the field, and the collaboration between traditional birth attendants and midwives | Development of an inclusive framework for collaboration between midwives and traditional birth attendants for maternal and child healthcare |  | This paper will contribute to the development of an inclusive healthcare framework, providing insights for stakeholders, policymakers, and practitioners seeking to improve maternal and child healthcare outcomes in resource-constrained, rural settings. |
| 41 | Application to promote communication between midwives and Arabic-speaking women at antenatal care: Challenges met by researchers when developing content | Bitar et al.  2022 [65] | To describe challenges met by researchers in developing culturally sensitive content in a Swedish-Arabic App to support communication needs of midwives | A co-design methodology was used for the development of the App, entailing collaboration between users and researchers in five different phases: user needs and preferences, development, field testing I, refinement, and field testing II | Co-design | The cross-disciplinary research team included a midwifery researcher, an Arabic-speaking midwife, three Swedish language specialists, and a health informatics expert, collaborating with end-users and software developers. | A multimodal app |  | Communication is essential for good health and encompasses linguistic and cultural aspects. Developing App content is a complex process, it does not only require researchers’ knowledge of the subject but there are also many other factors to consider such as user-friendliness, norm criticism, and culture appropriateness |
| 42 | Co-creation of a toolkit to assist risk communication and clinical decision-making in severe preeclampsia: SPOT-Impact study design | Amoakoh et al.  2024  Ghana [66] | To co-create a tool(kit) to support clinical decision-making, communication of risks and shared decision-making in preeclampsia with relevant stakeholders, incorporating respectful  maternity care, justice, and equity principles  To assess barriers to, and facilitators of, meaningful participation in co-creation.  To assess understandings, preferences, and suitable forms of a) risk communication and b) SDM.  To assess personal, interpersonal and system barriers to, and facilitators of, SDM. | A mixed methods design, combining qualitative methods with quantitative data collection and analysis | Co-creation – Experience Based Co-design  PRODUCES framework | 120 participants, including policy makers (3), health providers including obstetricians and midwives (56), facility managers (2), line managers (5), patients in stable conditions (10), survivors of HDP (37), male partners (6) and a relative (1) | Development of the SPOT toolkit |  | The transdisciplinary SPOT-impact study seeks to enhance the quality of care for women with HDP in terms of both clinical care and experience of care by creating a tool(kit) that can support risk prediction, risk communication, and shared-decision making. |
| 43 | Co-Creation of Massive Open Online Courses to Improve Digital Health Literacy in Pregnant and Lactating Women | Álvarez-Pérez et al.  2022 [67] | To develop MOOCs oriented towards the improvement of the DHL of European PLW, using a co-creation process that makes the final product respond to the needs and interests of the targeted participants | The development of the MOOCs involved a literature review, an exploratory survey of IC-Health participants, focus groups, group interviews, and the formation of communities of practice (CoPs) to co-design the MOOCs. | Co-design | Each CoP was composed of key stakeholders (PLW, their partners, researchers and interested healthcare professionals as midwives and lactation monitors) and coordinated by a CoP coordinator | Massive Open Online Courses |  | The preliminary results of the quantitative assessment showed a higher self-perceived DHL after the IC-Health MOOCs. These results suggest that IC-Health MOOCs and the co-creation methodology appear to be a viable process to carry out an intervention aimed to improve DHL levels in European PLW |
| 44 | Codesign and refinement of an optimized antenatal education session to better inform women and prepare them for labour and birth | Merriel et al.  2024  England [68] | To codesign, implement, evaluate acceptability and refine an optimized antenatal education session to improve birth preparedness. | The methods are presented in the four phases: codesign (2019), trial implementation (2021–2022), evaluation and refinement (2021–2022). | Co-design | The team included obstetricians, midwives, psychologists, service users, NCT representatives, an epidemiologist, project manager, and medical students. Parents were involved from the start and played an active role in the design. Postnatal and antenatal women, birthing people, and birth partners participated in the intervention, with midwives invited to deliver it. | A structured ANE session about labour and birth | We report on whether the optimized session is deliverable, acceptable, meets the needs of women/birthing people and partners, and explain how the intervention was refined with input from parents, clinicians and researchers. | A structured ANE session about labour and birth that provides women/pregnant people with the information they want and need to prepare for birth, within the constraints of available NHS resource - This intervention was positively received by parents. |
| 45 | Co-designing a theory-informed intervention to increase shared decision-making in maternity care | Waddell et al.  2023  Australia [69] | To develop a series of interventions to increase the use of SDM in maternity care with stakeholders | Study design, data collection and analysis are based on the Consolidated Criteria for Reporting Qualitative Research (COREQ) | Co-design | Participants included six clinicians (obstetricians, midwives and allied health), six health service leaders, three health service administrators, two patients and one government policy maker. | Three interventions were developed: access to maternity records via a patient portal and EMR, a persuasive communications campaign, and SDM simulation training with clinical champions. |  | This study fills literature gaps by involving end users and stakeholders in the co-design process, offering guidance to health services transitioning from policy to practice in pain control during labor. |
| 46 | Development of a co-designed behaviour change intervention aimed at healthcare professionals recruiting to clinical trials in maternity care | Hanrahan et al.  2022 [70] | To use behavioral theory in a co-design process to develop an intervention aimed at changing the behaviour of MHCPs recruiting to clinical trials in maternity care and to assess the acceptability and feasibility of such an intervention. | A step-wise sequential mixed-methods approach | Co-design | Stakeholders include maternity healthcare professionals and maternity service users | Development of the ENCOUNTER app |  | We developed a behaviour change recruitment intervention which is based on the accounts of MHCP trial recruiters and developed in a co-design process. Overall, the intervention was deemed acceptable. |
| 47 | Identifying health literacy solutions for pregnant women and mothers in Tasmania: a codesign study | Melwania et al.  2023  Tasmania [71] | To codesign health literacy solutions for pregnant women and mothers in Tasmania, Australia with stakeholders working in maternal and child health services across Tasmania and to develop a set of recommendations to guide the implementation of identified solutions in the Tasmanian context | Qualitative codesign workshops were conducted online | Co-design | Public health professionals, healthcare providers, managers, and allied health pro­fessionals working in maternal and child health | Foster multisectoral collaboration with existing organizations to map and enhance current programs. Strengthen system-level support through responsive health systems, policy changes, and workforce capacity building. Empower women and future generations to advocate for their health. |  | The solutions encourage policymakers to carefully consider the health literacy needs of pregnant women and mothers and support effective engagement of relevant stakeholders in planning and designing fit-for-purpose health literacy solutions. |
| 48 | Incorporating co-design principles and social media strategies to enhance cross-sectional online survey participation: The Birth Experience Study | Keedle H et al.  2024  Australia [72] | To use co-design principles to design a nationwide maternity experiences-survey and to distribute the survey through social media | A co-designed, cross sectional, and national online survey | Co-design | This survey was co-designed with maternity users and maternity organizations to explore women's recent experiences of maternity care in Australia | A well-designed online survey |  | The outcomes of this survey will support greater understanding of the experiences of women accessing maternity care and inform the design of sustainable and effective maternity services in Australia |
| 49 | Moving through Motherhood: Involving the Public in Research to Inform Physical Activity Promotion throughout Pregnancy and Beyond | Salmon et al.  2021 [73] | To explore the physical activity experiences and needs of women during and after pregnancy.  To demonstrate the value of this collaborative process in the design of evidence-based, user-centred physical activity information and resources which reflect diverse experiences of pregnancy and early parenthood and to argue for the normalisation of such approaches in this field | Two iterative phases which are described in Figure 1: Phase I: (a) patient and public involvement (PPI) workshop 1, (b) online survey, and (c) pilot resource development 1; Phase II: (a) stakeholder event, (b) PPI workshop 2, (c) and pilot resource development 2 | Co-design | Academics, clinical HCPs, public health practitioners, physical activity service providers, government or local authority representatives, charity representatives, pregnant women and women with young children |  |  | Our findings emphasize the need for clear, credible, and relatable information. Collaborating with women and stakeholders highlighted the importance of understanding diverse experiences in communicating about physical activity. This project shows that a collaborative approach is essential for developing effective research methods and resources. |
| 50 | Redesigning antenatal care: Prospective use of an implementation framework to establish a population-based multidisciplinary first-trimester screening, assessment and prevention service | Foster et al.  2024  Australia [74] | To redesign antenatal care within an Australian public healthcare center to align with evidence-based maternity care, including population-based first-trimester screening with early initiation of preventative strategies in high-risk pregnancies | A five-phase action-process model, aligned with implementation science theory, was used to explore barriers in antenatal care, co-design a novel service with consumers, and establish a population-based antenatal pathway starting with a multidisciplinary first-trimester visit. | Co-design | The team included obstetricians, midwives, sonographers, general practitioners (GP), allied healthcare staff (administrative staff, social workers, Aboriginal health workers) and service consumers | Development of the multidisciplinary IMAP (Initial Maternity Assessment and Planning) Education package for women aligned with low-risk and high-risk antenatal care schedules and elements of care |  | Use of an implementation action-process model allowed redesign of a large Australian public maternity service to align with the principle of first-trimester screening and treatment of high-risk pregnancies to reduce adverse pregnancy outcomes. |
| 51 | The challenges and opportunities for implementing group antenatal care ('Pregnancy Circles') as part of standard NHS maternity care: A co-designed qualitative study | Wiseman et al.  2022 [75] | To identify the challenges and opportunities for rolling out a bespoke model of group antenatal care called Pregnancy Circles (PC) within the National Health Service: what kind of support and training is needed and what adaptations are appropriate, including during a pandemic when face-to-face interaction is limited | Exploratory qualitative study (online focus group) | Co-design | Study co-designed with midwives | Integrating Pregnancy Circles  Three themes operating across the ecological model were identified: 'Implementing innovation', 'Philosophy of care' and 'Resource management' |  | Midwives facilitating group care valued the relationships, autonomy, and professional development it offered. To ensure wider implementation, challenges at macro and meso-levels, such as temporal, practical, and cultural issues, must be addressed. The PC model may adapt to online delivery and extend continuity of care, though further research is needed. |
| 52 | The Co‑development and Feasibility‑Testing of an Innovative Digital Animation Intervention (DAISI) to Reduce the Risk of Maternal Sepsis in the Postnatal Period | Haith‑Cooper et al.  2020 [76] | To enhance postnatal women’s awareness of sepsis and how to reduce their risk of developing the condition | DAISI was developed over a six-month period, through an iterative co-design process with key stakeholders. | Co-design | A skilled facilitator guided discussion of different stakeholders including clinical midwives and women with infants (including women from BAME backgrounds) | The final version of DAISI is a 3 min animation comprising of a purple-colored female avatar dressed in clothing covering her hair and body  The animation includes subtitles and a voice over currently available in English and Urdu. |  | DAISI appears to be an innovative solution for use in maternity care to address difficulties with the postnatal hospital discharge process.  If effective, the DAISI principle could be used in other maternity contexts and other areas of the NHS to communicate health promotion information |
| 53 | The development of a digital intervention to increase influenza vaccination amongst pregnant women | Parsons et al.  2021 [77] | To increase the uptake of the flu vaccination amongst pregnant women by targeting risk and efficacy appraisals | Six predefined steps were performed in line with Intervention Mapping. | Co-design | The intervention was developed by behavioral scientists, pregnant women, midwives, clinicians and Public Health professionals | The animation informed pregnant women about flu transmission, increased risk during pregnancy, potential consequences for mother and baby, vaccine contents, administration, and how it protects against flu. |  | This is the first known intervention for pregnant women, aiming to increase flu vaccination through addressing risk and efficacy appraisals. It has been implemented within seasonal flu vaccination campaigns during 2018/19 and 2019/20 within one geographically and ethnically diverse area of the UK. |
| 54 | The development of an IT-based intervention to support a healthy postpartum lifestyle through behavioral change | Christiansen et al.  2023 [78] | To provide new insights into developing and designing information and communication technology interventions to support a healthy postpartum lifestyle through behavioral changes | A participatory design approach, combined with the behavior change wheel, was applied | Co-creation | Stakeholders included postpartum parents, healthcare professionals, IT consultants, and researchers | The intervention includes podcasts, video exercises, weight tracking, and weekly push notifications |  | Using participatory design and the behavior change wheel, we successfully designed and developed an intervention that seeks to meet users’ needs and suggests behavioral change techniques |
| 55 | Using Intervention Mapping to Develop a Workplace Digital Health Intervention for Preconception, Pregnant, and Postpartum Women: The Health in Planning, Pregnancy and Postpartum (HiPPP) Portal | Blewitt et al.  2022  Australia [79] | To describe the use of the IM methodology to develop a workplace digital health intervention to promote healthy lifestyles and wellbeing for PPP working women; our partner workplace is a community service organisation. | IM was adopted due to its focus on behaviour change, guidance for integrating theory and research evidence, co-design approach with those who will use and implement the program, and extensive use in practice | Co-design | Staff from MacKillop with appropriate expertise or lived experience participated in the design group. This included women aged 18–45 years (given the focus on PPP women) | Development of the HiPPP Portal – A digital health intervention that has been specifically designed and tailored to the workplace and needs of PPP women |  | Findings from a pilot feasibility study indicate the portal was accessible and beneficial for women in PPP life stages. The Intervention Mapping protocol may offer a valuable roadmap for collaborative design of interventions targeting PPP women’s behavior and organizational work culture. |
| 56 | Using the Consolidated Framework for Implementation Research to design and implement a perinatal education program in a large maternity hospital | Guyatt et al.  2021  Australia [80] | To outline the synthesis of a theoretical, multidimensional implementation science approach to delivering an effective perinatal education intervention | Implementation science provides a theoretical approach to design and implement perinatal care interventions | Co-design | Both expectant and new parents and their health care providers who develop and deliver perinatal education |  |  | This multidimensional theoretical approach provides useful, practical guidance to health service researchers and clinicians to develop project specific rationale for their theoretical approach to implementation projects |
| 57 | Co-creation to scale up provision of simplified high-quality comprehensive abortion care in East Central and Southern Africa | Klingberg-Allvin et al.  2018 [81] | To address the issue of unsafe abortion and limited CAC access in the region  To identify both barriers to and facilitators of implementing CAC in East Africa  To facilitate improvements in the quality of CAC and to create discussions about co-creation, exchange, and capacity building in ECSA to improve access to CAC  To create a network consisting of researchers and abortion care providers in Africa similar to FIAPAC |  | Co-creation | Workshop participants included Swedish and ECSA scientific experts in CAC, health ministry representatives, FIGO's unsafe abortion prevention group, NGOs, and media partners | Multiple workshops | The outcome of the workshop was the identification of sustainable solutions to improve CAC in the region:  Focus on local capacity in building in-service training and education,  Ensure that curricula are evidence based and updated  Include value clarification and the use of simple, evidence-based procedures for CAC | This type of infrastructure for exchanging information and developing co-creation could be crucial to advancing the Sustainable Development Goals 2030 agenda |
| 58 | “They just looked at me like I was human”: The experiences of parenting women and providers with substance use disorder treatment | Bosak et al.  2024  United States of America [82] | To identify barriers and facilitators to engagement and retention in SUD residential treatment for pregnant and parenting women | This manuscript draws on findings from qualitative interviews collected during the first stage of an adapted EBCD process to develop a residential treatment program specifically for pregnant and parenting women to attend with their children. | Co-design | Both parenting women with lived experience (WWLE) in residential SUD treatment and SUD treatment providers |  |  | This research increases understanding of the interplay of the structural and relational barriers and facilitators to engagement and retention in treatment. These seemingly minor positive or negative interactions along the care continuum are pivotal to fully operationalizing TIC and optimizing women's engagement in treatment. |
| 59 | A consumer co-created infographic improves short-term knowledge about physical activity and self-efficacy to exercise in women with gestational diabetes mellitus: a randomized trial | Harrison et al.  2020  Australia [83] | To determine whether addition of a consumer co-created infographic to usual education about gestational diabetes mellitus improve knowledge about physical activity and self-efficacy to exercise | A randomized trial with concealed allocation, a blinded assessor and intention-to-treat analysis. | Co-creation | Consumers - 13 pregnant women with GDM who consented to participate in a 20-minute, face-to-face, group consultation meeting | The experimental group received standard gestational diabetes education along with a consumer co-created infographic on physical activity during gestational diabetes, while the control group received only the standard education. | Participants completed baseline and follow-up measures after one week. Knowledge of physical activity during gestational diabetes was assessed with a 19-item questionnaire, and self-efficacy with the 9-item Self-Efficacy for Exercise Scale. | This study found that adding a co-created infographic to usual GDM education improved short-term knowledge and self-efficacy for physical activity during GDM pregnancy. |
| 60 | Co‐designing The Healthy Gut Diet for Preventing Gestational Diabetes: Co‐design methods and process outcomes | Meloncelli et al.  2024 [84] | To report on the co-design process used to develop The Healthy Gut Diet intervention | Co-designing The Healthy Gut Diet intervention used qualitative approaches most closely aligned to Participatory Action Research | Co-design | Consumer participants (women with a lived experience of GDM), researchers and workshop co-facilitators | Development of a dietary intervention – The Healthy Gut diet |  | The Healthy Gut Diet trial is a co-designed study for pregnant women at risk of GDM, developed with input from women with lived experience. It follows MRC guidelines, detailing methods and outcomes to ensure the intervention is reproducible and adaptable, using the TIDieR checklist. |

Supplementary file 1 – Characteristics of the included studies (n=60)
